# Sls1 and Mtf2 mediate the assembly of the Mrh5C complex required for activation of *cox1* mRNA translation

**DOI:** 10.1016/j.jbc.2024.107176

**Published:** 2024-03-16

**Authors:** Yirong Wang, Ting Jin, Ying Huang

**Affiliations:** Jiangsu Key Laboratory for Microbes and Functional Genomics, Nanjing Normal University, Nanjing, China

**Keywords:** *Schizosaccharomyces pombe*, mitochondrial translation, *cox1* mRNA, oxidative phosphorylation, PPR protein

## Abstract

Mitochondrial translation depends on mRNA-specific activators. In *Schizosaccharomyces pombe*, DEAD-box protein Mrh5, pentatricopeptide repeat (PPR) protein Ppr4, Mtf2, and Sls1 form a stable complex (designated Mrh5C) required for translation of mitochondrial DNA (mtDNA)-encoded *cox1* mRNA, the largest subunit of the cytochrome *c* oxidase complex. However, how Mrh5C is formed and what role Mrh5C plays in *cox1* mRNA translation have not been reported. To address these questions, we investigated the role of individual Mrh5C subunits in the assembly and function of Mrh5C. Our results revealed that Mtf2 and Sls1 form a subcomplex that serves as a scaffold to bring Mrh5 and Ppr4 together. Mrh5C binds to the small subunit of the mitoribosome (mtSSU), but each subunit could not bind to the mtSSU independently. Importantly, Mrh5C is required for the association of *cox1* mRNA with the mtSSU. Finally, we investigated the importance of the signature DEAD-box in Mrh5. We found that the DEAD-box of Mrh5 is required for the association of Mrh5C and *cox1* mRNA with the mtSSU. Unexpectedly, this motif is also required for the interaction of Mrh5 with other Mrh5C subunits. Altogether, our results suggest that Mrh5 and Ppr4 cooperate in activating the translation of *cox1* mRNA. Our results also suggest that Mrh5C activates the translation of *cox1* mRNA by promoting the recruitment of *cox1* mRNA to the mtSSU.

Mitochondria are eukaryotic organelles that generate cellular energy in the form of ATP *via* oxidative phosphorylation (OXPHOS) (1). In addition, mitochondria play a key role in many other cellular processes, including apoptosis, signaling, the metabolism of lipids and amino acids, and the synthesis of iron-sulfur clusters and heme (1, 2, 3).

Mitochondria have retained their own genome (mtDNA) which primarily encodes core catalytic subunits of OXPHOS complexes, as well as the full set of tRNAs and two rRNAs required for mitochondrial translation. In *Saccharomyces cerevisiae* and *Schizosaccharomyces pombe*, mtDNA encodes seven core subunits of OXPHOS complexes III (ubiquinol-cytochrome *c* reductase complex or cytochrome *b-c1* complex), IV (cytochrome *c* oxidase) and V (ATP synthase), and a component of the small subunit of the mitochondrial ribosome (mitoribosome) (Var1, also called Rps3). Once synthesized, mtDNA-encoded OXPHOS subunits initiate the assembly of their respective complexes.

Translation of mtDNA-encoded mRNAs (mt-mRNAs) requires mRNA-specific activators (4, 5, 6, 7). In *S. cerevisiae*, translation of mtDNA-encoded complex IV subunit Cox1 requires the concerted action of factors including Mss51, the pentatricopeptide repeat (PPR) protein Pet309, Mam33, DEAD-box helicase Mss116. Pet309 is suggested to bind to the 5′-untranslated region (5′-UTR) of the *cox1* mRNA and recruits Mss116, which unfolds the *cox1* mRNA secondary structures (8). Mss51 couples the *cox1* mRNA translation and assembly of complex IV (9). Translation of complex III subunit Cob1 (also called COB or Cytb) involves Cbp1, required for *cob1* mRNA stability, Cbs1 and Cbs2, which bind *cob1* 5′-UTR and mitoribosome, and the Cbp3-Cbp6 complex, which like Mss51, coordinates *cob1* mRNA translation and the subsequent assembly of complex III (10). The Cbp3-Cbp6 complex activates Cob1 translation by displacing Cbs1 from the mitoribosome exit tunnel and supports hemylation of newly synthesized Cob1 (10). More importantly, this complex participates in feedback regulation of Cob1 translation by dissociating from nascent Cob1 upon Qcr8 association and recycling back to the mitoribosome for use in further rounds of Cob1 translation (11). It has been found that Mrh5, Ppr4, Sls1, and Mtf2 form a protein complex (hereafter called Mrh5C) specifically required for *cox1* mRNA translation in *S. pombe* (12). In humans, TACO1 and complex IV assembly factors MITRAC and C12orf62 specifically stimulate *COX1* mRNA translation (13, 14).

The requirement for mt-mRNA-specific translational activators is due in part to the fact that the 5′-UTRs of mt-mRNAs lack a canonical Shine-Dalgarno (SD) sequence, which serves as a ribosome-binding site and is essential for translational initiation in bacteria. Indeed, it has been shown that *S. cerevisiae* mt-mRNA-specific translational activators are likely to play a role in the translation initiation of mt-mRNAs by acting on the 5′-UTRs of target mt-mRNAs (8, 15, 16). However, the mechanisms involved remain largely unknown. In contrast, mammalian mt-mRNAs lack 5′-UTR, and thus, it is generally believed that mammals use different mechanisms for translation initiation (13, 17).

Mitochondrial translation also requires general translational activators. In *S. cerevisiae*, Sls1 and Mtf2 (also called Nam1) are required for translation of all mtDNA-encoded transcripts (18). The two proteins are likely to function together to coordinate mitochondrial translation and transcription. In *S. pombe*, Ppr2 (19) and the Ppr10-Mpa1 complex (20, 21, 22) are globally required for mitochondrial protein synthesis. In humans, LRPPRC (23, 24, 25), IGF2BP1 (26), and DHX30 (26) are involved in the translation of mt-mRNAs.

PPR proteins are involved in post-transcriptional processes of organellar genes, including RNA 5′-end maturation, intron splicing, RNA editing and RNA stabilization, and mitochondrial translation (27, 28, 29, 30). PPR proteins are characterized by 2 to 30 tandem repeats of a degenerate ∼35-amino acid (aa) sequence repeat. PPR repeats are involved in sequence-specific RNA binding, with each repeat recognizing a single nucleotide (31). In addition, they also participate in protein–protein interactions in humans (32). So far, several PPR proteins, including *S. cerevisiae* Pet111 (15) and Pet309 (33), *S. pombe* Ppr2 (19) and Ppr10 (20, 21, 22), and human LRPPRC (23, 24, 25), have been identified to be required for mitochondrial translation.

DEAD-box proteins contain a helicase domain that comprises two RecA-like helicase subdomains (D1 and D2) connected by a short flexible linker and is characterized by a set of conserved motifs including the signature DEAD motif. The DEAD motif is a variant Walker B motif and is responsible for RNA binding and unwinding in a sequence-independent manner as well as ATP hydrolysis. DEAD-box proteins are involved in all aspects of RNA metabolism: intron splicing, mRNA export and decay, ribosome biogenesis, and translation (34, 35). So far, only a handful of DEAD-box proteins involved in mitochondrial translation have been identified. These include Mss116 (8), Irc3 (36), Mrh5 (12) and DHX30 (26). However, their exact roles in mitochondrial translation remain unclear.

In this study, we investigate each subunit’s role in the assembly and function of Mrh5C. We demonstrate that Sls1 and Mtf2 are required for the assembly of Mrh5C, whereas Mrh5 and Ppr4 play minor roles. Mrh5C binds to both the small subunit of the mitoribosome (mtSSU) and *cox1* mRNA, and is required for the association of *cox1* mRNA with mtSSU. Our work provides insights into how Mrh5C activates *cox1* mRNA translation.

## Results

### Sls1 and, to a lesser extent, Mrh5 are required for the stability of Mtf2

**Figure 1:**
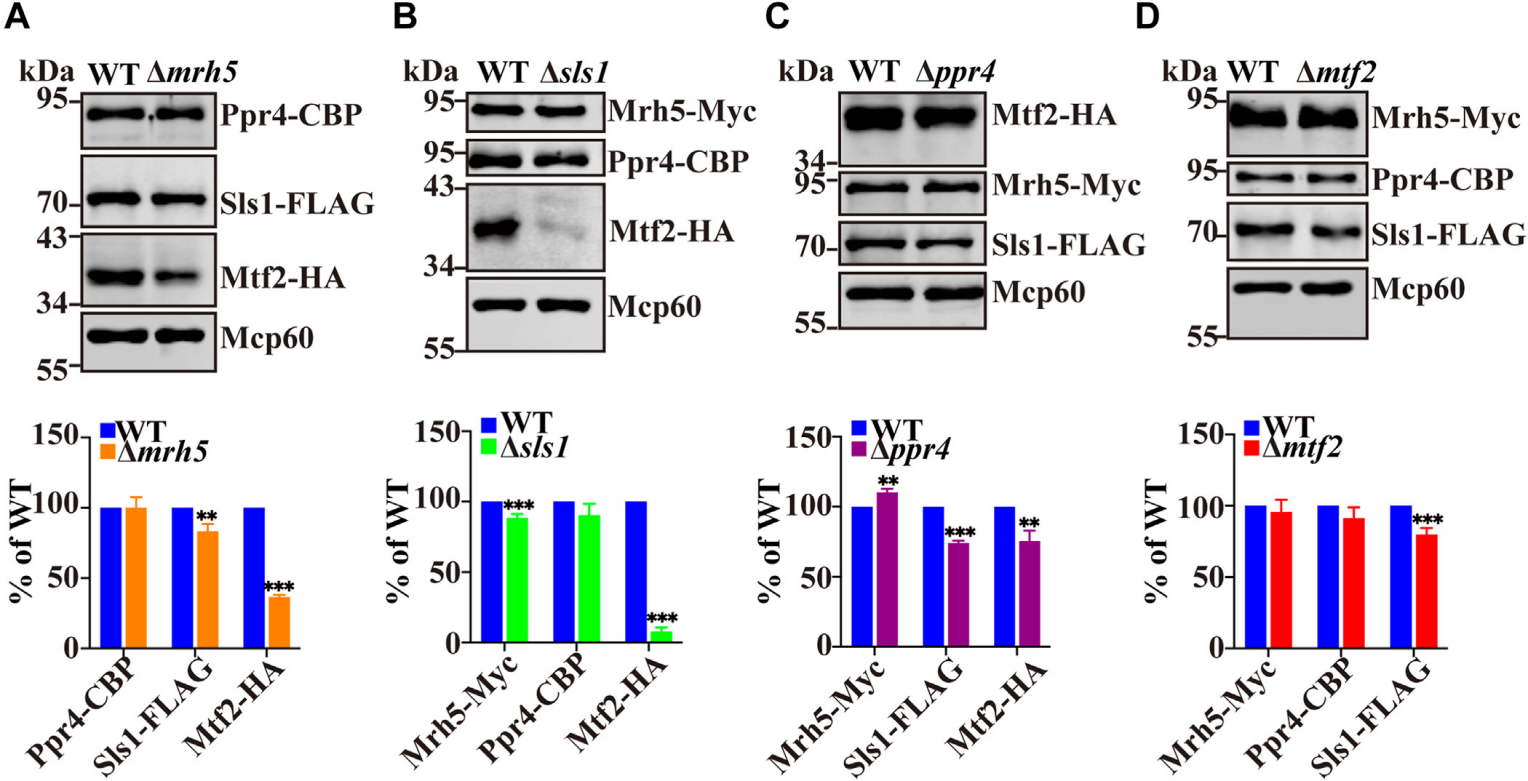
**Sls1and to a lesser extent,Mrh5 are required for the stability ofMtf2.** Overnight cultures of Δ*mrh5* (*A*), Δ*sls1* (*B*), Δ*ppr4* (*C*), and Δ*mtf2* (*D*) cells were diluted in fresh YES to an OD_600_ of 0.2 and continued to grow to log phase. Cells were harvested, and mitochondria were prepared using the spheroplast method. Mitochondrial extracts were analyzed by immunoblotting using indicated Abs. Protein levels were quantified and expressed as percentage change with respect to WT. Data were normalized to Mcp60 and presented as mean ± S.D. of three independent experiments. Statistically significant differences were determined by Student′s *t* test (∗, *p* < 0.05; ∗∗, *p* < 0.01; ∗∗∗, *p* < 0.001).

It has been suggested that *S. pombe* Mrh5 and Ppr4 are functionally analogous to *S. cerevisiae* Mss116 and Pet309, respectively (12). Mss116 is required for the stability of Pet309. To investigate if this holds true for Mrh5 and Ppr4, we monitored the Ppr4 protein level in Δ*mrh5* cells by immunoblotting. Deletion of *mrh5* did not reduce the protein level of Ppr4 (Fig. 1*A*). We next examined whether the stability of Mrh5C subunits is affected by their interactions with other subunits. To this end, we individually deleted each subunit of Mrh5C and monitored the protein levels of the remaining subunits in the resulting deletion mutants. To facilitate the detection of Mrh5C subunits, we endogenously tagged Mrh5, Ppr4, Sls1, and Mtf2 with Myc, calmodulin-binding peptide (CBP), FLAG, and hemaglutinin (HA), respectively. The functions of these proteins are apparently not affected by the presence of the respective tag, as assayed by growth on the respiratory carbon source glycerol (Fig. S1). Deletion of *sls1* resulted in a drastic reduction in the protein level of Mtf2 (Fig. 1*B*). Additionally, the Mtf2 protein level was moderately reduced in Δ*mrh5* cells (Fig. 1*A*). In contrast, the deletion of *ppr4* and *mtf2* did not or only moderately affect the protein levels of other Mrh5C subunits (Fig. 1, *C* and *D*).

### Mrh5C formation was virtually abolished by deletion of *mtf2* or *sls1*

**Figure 2:**
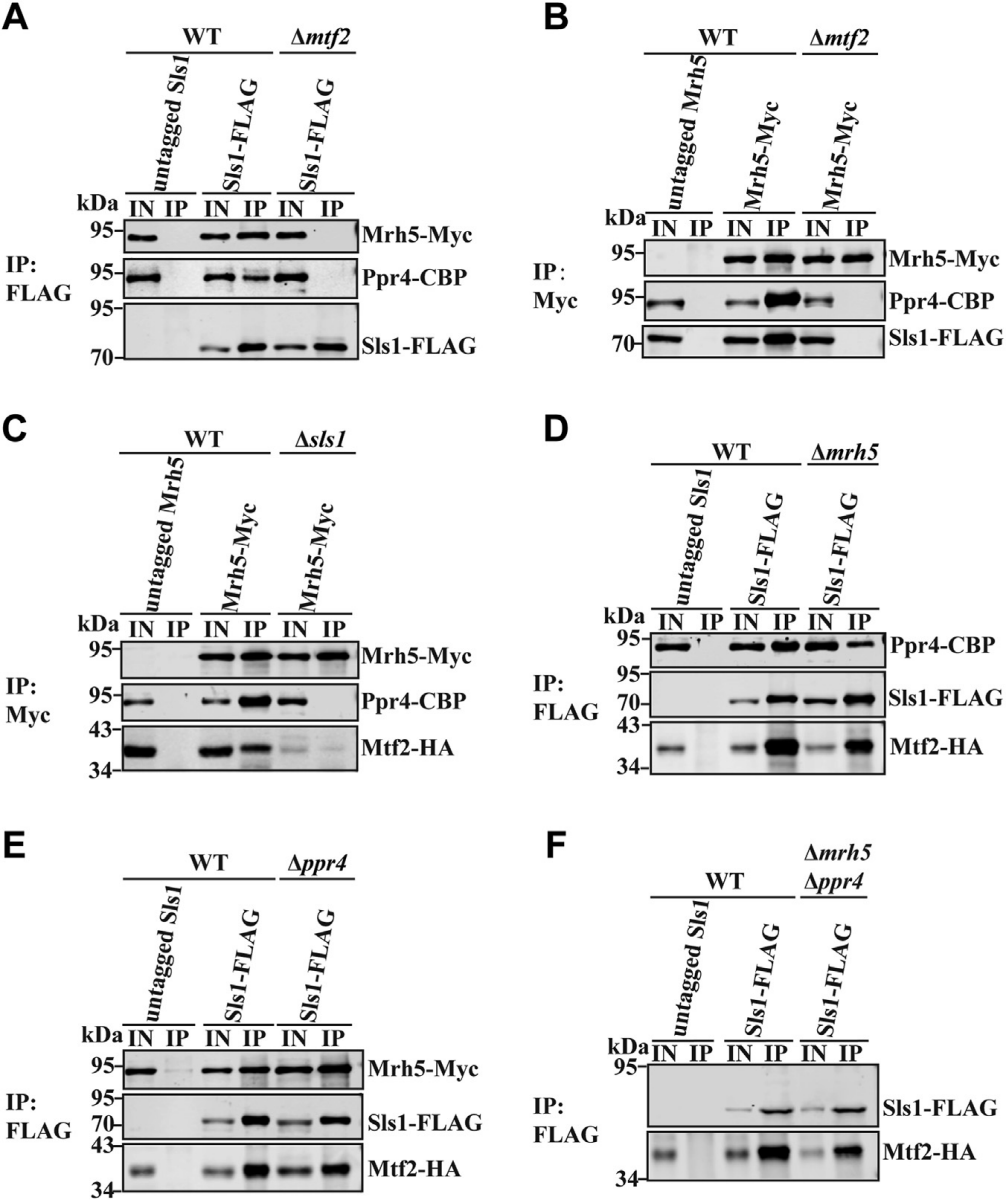
**Mtf2 and Sls1 are required for the assembly of Mrh5C.***A* and *B*, loss of Mtf2 disrupts Mrh5C. Mitochondrial extracts prepared from WT cells expressing untagged Sls1 (negative control) or Sls1-FLAG (positive control), and Δ*mtf2* cells expressing Sls1-FLAG were subjected to anti-FLAG co-immunoprecipitation (*A*). Mitochondrial extracts prepared from WT cells expressing untagged Mrh5 or Mrh5-Myc, and Δ*mtf2* cells expressing Mrh5-Myc were subjected to anti-Myc co-immunoprecipitation (*B*). *C*, loss of Sls1 disrupts Mrh5C. WT cells expressing untagged Mrh5 or Mrh5-Myc and Δ*mtf2* cells expressing Mrh5-Myc were subjected to anti-Myc co-immunoprecipitation. *D*, loss of Mrh5 does not affect interactions among other members of Mrh5C. WT cells expressing untagged Sls1 or Sls1-FLAG, and Δ*mrh5* cells expressing Sls1-FLAG were subjected to anti-FLAG co-immunoprecipitation. *E*, loss of Ppr4 does not affect interactions among other members of Mrh5C. WT cells expressing untagged Sls1 or Sls1-FLAG, and Δ*ppr4* cells expressing Sls1-FLAG were subjected to anti-FLAG co-immunoprecipitation. *F*, Mtf2 and Sls1 form a complex independently of Mrh5 and Ppr4. WT cells expressing untagged Sls1 or Sls1-FLAG, and Δ*mrh5*Δ*ppr4* cells expressing Sls1-FLAG were subjected to anti-FLAG co-immunoprecipitation. Mitochondrial extracts (IN) and immunoprecipitates (IP) were analyzed by immunoblotting using specific anti-tag Abs. Genes encoding the Mrh5C subunits were endogenously tagged to facilitate their detection.

To determine how Mrh5C is formed, we individually deleted each subunit of Mrh5C and monitored the complex formation by co-immunoprecipitation. For co-immunoprecipitation assays, we endogenously tagged Mrh5, Ppr4, Sls1, and Mtf2 with Myc, CBP, FLAG, and HA, respectively. We first examined the effect of *mtf2* deletion on the formation of Mrh5C. Co-immunoprecipitation experiments with anti-FLAG beads showed that deletion of *mtf2* abolished the interaction of Sls1-FLAG with Mrh5-Myc and Ppr4-CBP (Fig. 2*A*). Co-immunoprecipitation experiments with anti-Myc beads revealed that the interaction between Mrh5-Myc and Ppr4-CBP was disrupted in the absence of *mtf2* (Fig. 2*B*). Next, we examined the effect of *sls1* deletion on Mrh5C formation. Co-immunoprecipitation experiments using anti-Myc beads revealed that loss of *sls1* abolished the association of Mrh5-Myc with Prp4-CBP and Mtf2-HA (Fig. 2*C*). Because the protein level of Mtf2 is dramatically reduced in the absence of Sls1, the effect of *sls1* deletion on Mrh5C is more likely to be indirect through Mtf2 destabilization. Deletion of *mrh5* did not impair the interaction among Prp4-CBP, Sls1-FLAG, and Mtf2-HA (Fig. 2*D*). Similarly, deletion of *ppr4* did not impair the interaction among Mrh5-Myc, Sls1-FLAG, and Mtf2-HA (Fig. 2*E*). Deletion of *mrh5* and *ppr4* did not impair the interaction between Mtf2-HA and Sls1-FLAG (Fig. 2*F*). Altogether, these results reveal that Mtf2 and Sls1 form a subcomplex that holds Mrh5 and Ppr4 together.

### The association of Mrh5C with the mtSSU requires the participation of all four subunits

**Figure 3:**
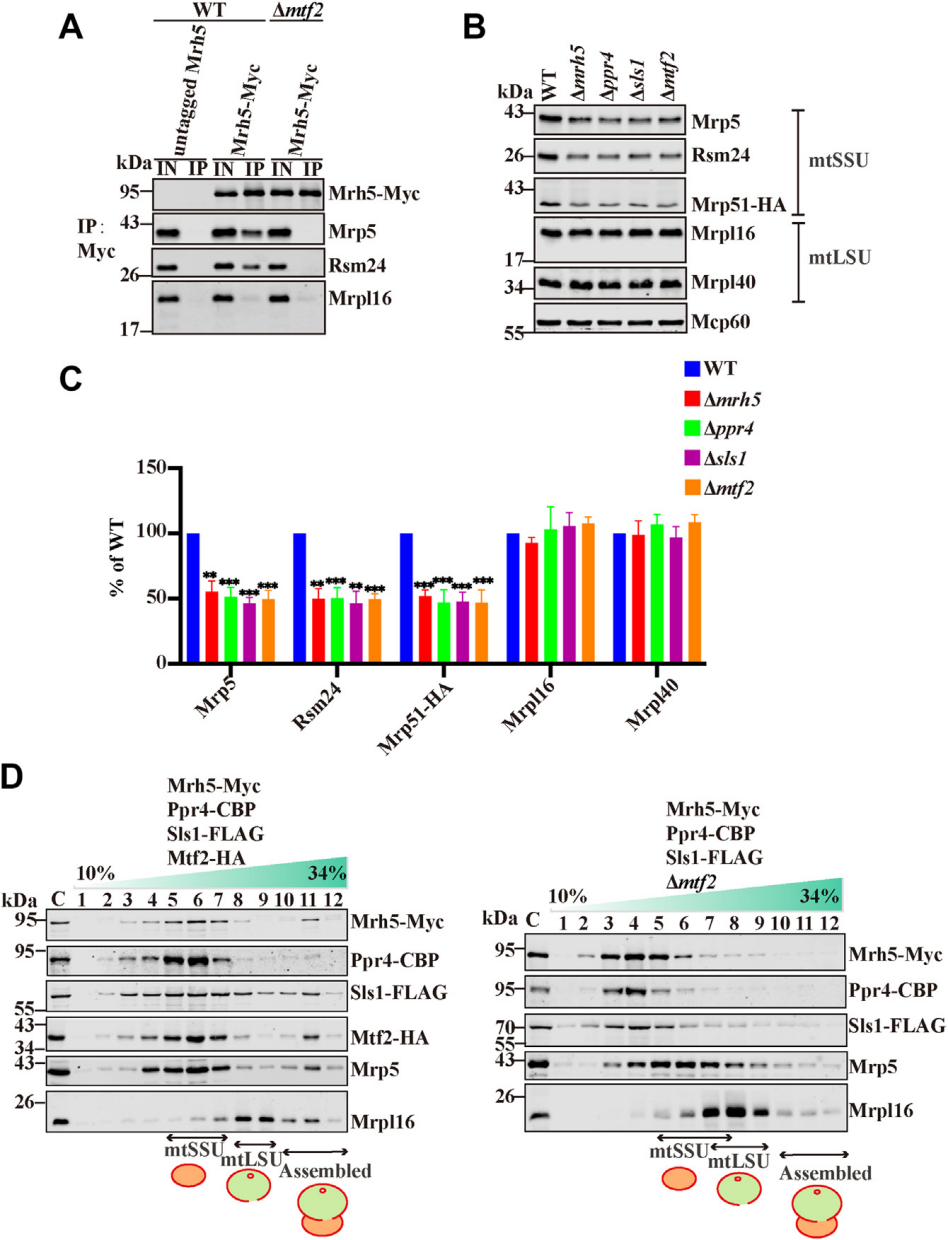
**The presence of all four subunits is required for binding of Mrh5C with the mtSSU.***A*, loss of Mtf2 impairs Mrh5 association with the mtSSU. WT cells expressing untagged Mrh5 or Mrh5-Myc, and Δ*mtf2* cells expressing Mrh5-Myc were grown to log phase. Mitochondrial extracts were prepared by the spheroplast method and used for anti-Myc co-immunoprecipitation. Mitochondrial extracts (IN) and immunoprecipitates (IP) were analyzed by immunoblotting for the indicated proteins. *B*, loss of subunits of Mrh5C reduces the levels of the mtSSU proteins but not the mtLSU proteins tested. WT, Δ*mrh5*, Δ*ppr4*, Δ*sls1* and Δ*mtf2* cells were grown to log phase. Mitochondrial extracts were prepared by the spheroplast method and subjected to immunoblotting for indicated proteins. *C*, quantitation of data in B was performed as described in Fig. 1, *D*, disruption of Mrh5C results in its dissociation from the mtSSU. Mitochondria isolated from WT cells expressing Mrh5-Myc, Ppr4-CBP, Sls1-FLAG and Mtf2-HA (*left panels*), and Δ*mtf2* cells expressing Mrh5-Myc, Ppr4-CBP, and Sls1-FLAG (*right panels*) were subjected to 10%–34% sucrose density gradient centrifugation. After collecting fractions (1, *top*; 12, *bottom*), proteins were TCA precipitated, and analyzed by immunoblotting. The mtSSU protein Mrp5 and the mtLSU protein Mrpl16 indicate the distribution of mitoribosomal complexes. C, total mitochondrial proteins.

It has been shown that Mrh5 co-purifies with the mitoribosomal proteins, suggesting that Mrh5C associates with the mtSSU (12, 37). However, it is unclear how the individual subunits of Mrh5C contribute to this association. To determine the subunit requirements for the association of Mrh5C with the mtSSU, we took advantage of the Δ*mtf2* deletion mutant in which Mrh5C subunits no longer associate with each other. Mrh5 co-immunoprecipitated with the mtSSU as expected (Fig. 3*A*). Deletion of *mtf2* dramatically reduced this association, suggesting that the whole complex is involved in mtSSU binding (Fig. 3*A*). Deletion of *mtf2* or other subunits of Mrh5C dramatically reduced the levels of mtSSU proteins but not the large subunit of the mitoribosome (mtLSU) proteins tested (Figure 3, *B* and *C*), suggesting that blocking *cox1* mRNA translation by disruption of Mrh5C could lead to destabilization of the mtSSU.

To more accurately evaluate the association of Mrh5C with the mitoribosome, mitoribosomal components from wild-type (WT), Δ*mtf2*, Δ*mrh5,* and Δ*ppr4* cells were separated by sucrose gradient ultracentrifugation and fractions analyzed by immunoblot analysis. Mrh5C was predominantly associated with the mtSSU in WT cells (Fig. 3*D*). In contrast, Mrh5C subunits no longer co-sedimented with the mtSSU in Δ*mtf2* cells (Fig. 3*D*). In addition, deletion of *mtf2* caused a reduction in the mitoribosome level, suggestive of impaired mitoribosome assembly in Δ*mtf2* cells. Similar results were obtained with Δ*mrh5* and Δ*ppr4* cells (Fig. S2). Altogether, these results further suggest that the Mrh5C subunits do not stably associate with the mtSSU unless they form a stable complex and that disruption of Mrh5C impairs mitoribosome assembly.

### Disruption of Mrh5C abolished the association of *cox1* mRNA with the mtSSU and mitoribosome

**Figure 4:**
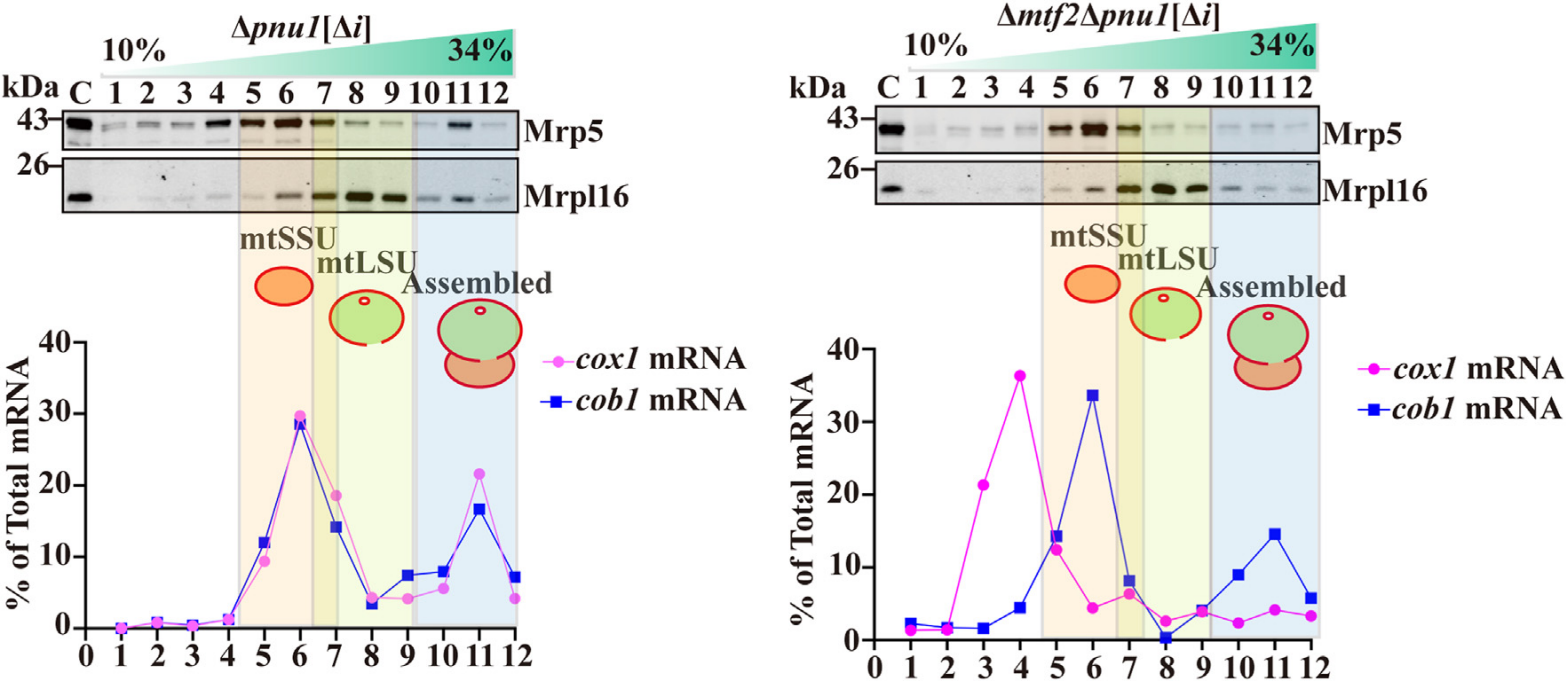
**Disruption of Mrh5Cformation abolishes the association of *cox1* mRNA with the mtSSUand mitoribosome.** Mitochondrial extracts were prepared from mtDNA intronless Δ*pnu1* (Δ*pnu1*[Δ*i*]) cells (*left panels*) and mtDNA intronless Δ*mtf2*Δ*pnu1* (Δ*mtf2*Δ*pnu1*[Δ*i*]) cells (*right panels*), followed by centrifugation on sucrose gradients. The positions of the mitoribosomal complexes on sucrose gradients were determined by immunoblotting using anti-Mrp5 and anti-Mrpl16 Abs (*upper panels*). The distribution of the *cox1* and *cob1* mRNAs on sucrose gradients was analyzed by qRT-PCR (*lower panel*). The peak fractions of the mtSSU, mtLSU, and assembled mitoribosome are marked by transparent *orange*, *green*, and *blue* colors, respectively. The data are expressed as a percentage of total specific RNA and are representative of three independent experiments.

Because Mrh5C specifically associates with *cox1* mRNA (37), we examined whether Mrh5C plays a role in the recruitment of *cox1* mRNA to the mtSSU. To this end, we performed sucrose gradient sedimentation of mitochondrial extracts from the WT[Δ*i*] strain and its isogenic Δ*mtf2* mutant (Δ*mtf2*[Δ*i*]), both of which carry a null allele of *pnu1*, encoding a mitochondrial endonuclease to minimize RNA degradation during sample preparation. It has been shown that the deletion of *nuc1* encoding the *S. cerevisiae* homolog of Pnu1 can prevent the degradation of mtDNA-encoded RNAs (38). Deletion of *pnu1* does not produce any respiratory phenotypes (Fig. S3). We chose strains carrying intronless mtDNA because deletion of *mtf2* in cells containing mtDNA introns resulted in a dramatic reduction in the levels of the mature *cox1* mRNA, and removal of the introns of mtDNA could restore the level of *cox1* mRNA to WT levels (39). After sucrose gradient centrifugation, about two-thirds of each fraction was used for RNA extraction and the levels of *cox1* and *cob1* mRNAs in each fraction were determined by qRT-PCR. The remaining one-third was used for immunoblotting. Mrp5 and Mrpl16 were used as markers for the mtSSU and mtLSU, respectively. In control cells (Δ*pnu1*[Δ*i*]), the *cox1* and *cob1* mRNAs appeared in two peaks, a major peak (peak A) comigrating with the mtSSU and a second peak (peak B) comigrating with the mitoribosome (Fig. 4). In Δ*mtf2* cells, peak A was up-shifted to the lower-density regions and did not comigrate with the mtSSU, and peak B decreased dramatically (Fig. 4). As a control, the association of *cob1* mRNA with the mitoribosome was not affected in Δ*mtf2* cells (Fig. 4). Similar results were obtained with Δ*mrh5* and Δ*ppr4* cells (Fig. S4). These results suggest that Mrh5C is required for the association of *cox1* mRNA with the mtSSU and mitoribosome.

### Mutations in the DEAD-box of Mrh5 primarily impair Cox1 synthesis

**Figure 5:**
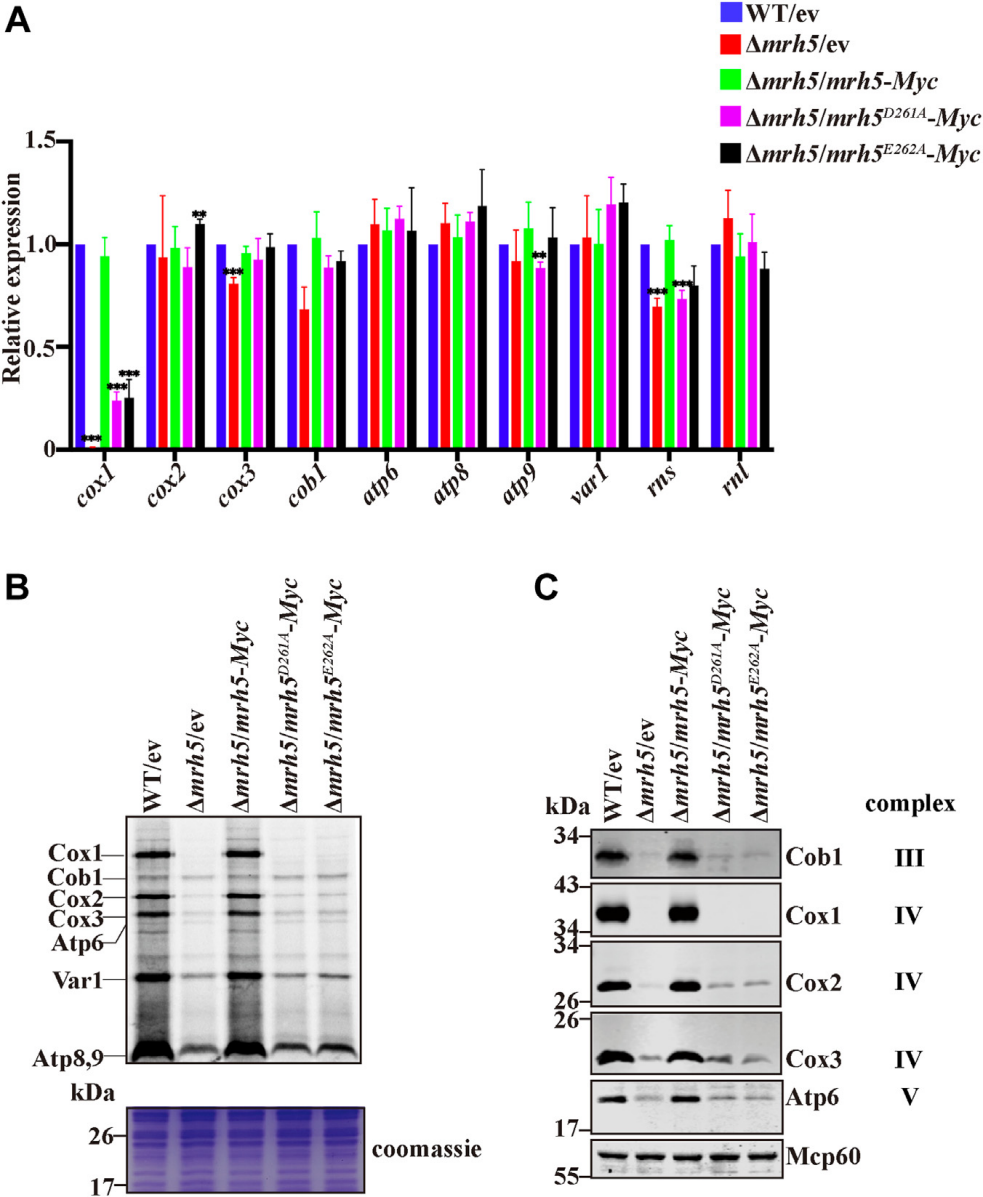
**The DEAD-box is required for Mrh5 function in mitochondrial translation.***A*, mutations in the DEAD-box of Mrh5 dramatically reduce the levels of *cox1* mRNA. Total RNA was isolated from WT bearing the empty vector (ev), Δ*mrh5* bearing ev and Δ*mrh5* cells bearing integrated *mrh5-Myc*, *mrh5*^*D261A*^*-Myc*, or *mrh5*^*E262A*^-*Myc* at the *leu1-32* locus under the regulation of its own promoter, and expression of mt-mRNA and mt-rRNA was measured by qRT-PCR. Results were normalized to *act1* mRNA. Values represent the mean ± S.D. of at least three independent experiments. Statistically significant differences were determined by Student′s *t* test (∗∗, *p*  <  0.01; ∗∗∗, *p*  <  0.001). *B*, mutations in the DEAD-box of Mrh5 impair global mitochondrial translation, particularly of Cox1. Mitochondrial translation products were labeled with [^35^S]-methionine/cysteine and analyzed by SDS/PAGE and autoradiography. *C*, mutations in the DEAD-box of Mrh5 reduce the steady-state levels of mtDNA-encoded protein, particularly of Cox1. Mitochondrial extracts were analyzed by SDS/PAGE and immunoblotting with Abs against mtDNA-encoded proteins. Mitochondrial matrix protein Mcp60 recognized by anti-HSPD1 Ab serves as a loading control.

We next chose to examine the functional importance of the DEAD-box of Mrh5. Because the first two negatively charged residues in the DEAD-box are conserved in all DEAD-box helicases and were shown to be essential for ATP-dependent RNA unwinding activity (40), we constructed two mutated *mrh5* alleles in which the first two residues (Asp261 and Glu262) in the DEAD-box were individually mutated to Ala. Each of the mutant alleles was integrated into the *leu1-32* locus of the Δ*mrh5* strain. A single Asp261 to Ala or Glu262 to Ala mutation gave rise to a substantially reduced level of *cox1* mRNA but little or no reduction of the levels of other mt-mRNAs and mt-rRNAs (Fig. 5*A*). These mutations also completely abolished Cox1 synthesis and decreased the synthesis of other mtDNA-encoded proteins (Fig. 5, *B* and *C*). It is likely that the abolishment of Cox1 synthesis results in the downregulation of OXPHOS complexes. Consistent with these results, cells harboring *mrh5*^*D261A*^-*Myc* or *mrh5*^*E262A*^-*Myc* could not grow on glycerol-containing medium, suggesting that they are respiratory-deficient (Fig. S5).

**Figure 6:**
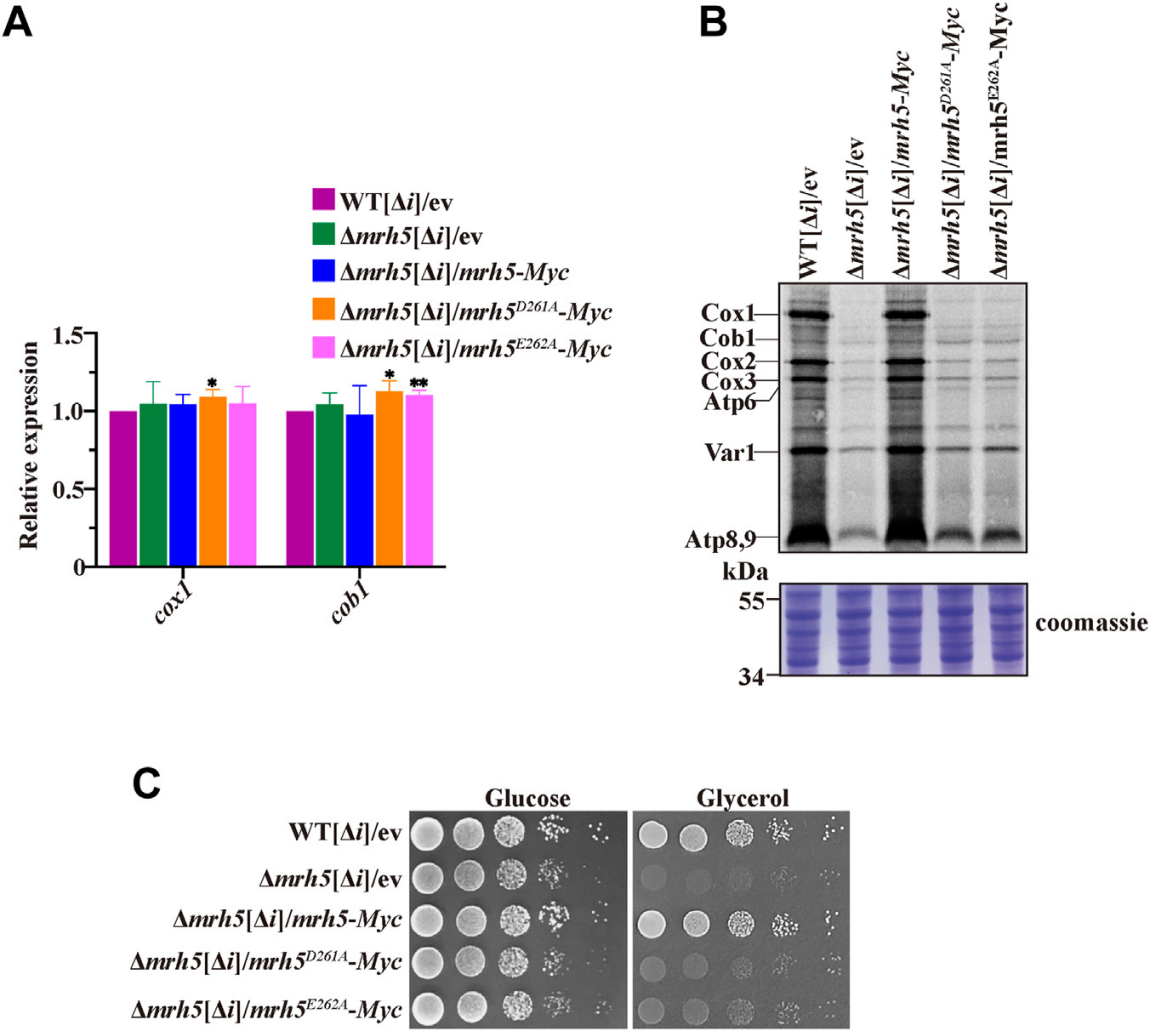
**Removal of mtDNA introns cannot rescue respiratory defect of cells harboring mutations in the DEAD-box of Mrh5.***A*, removal of the mtDNA introns restores the level of *cox1* mRNA in cells harboring mutations in the DEAD-box of Mrh5 to a level not significantly different from those of WT cells. Total RNA was isolated from WT[Δ*i*] cells bearing ev, and Δ*mrh5*[Δ*i*] cells harboring integrated ev, *mrh5*-*Myc*, *mrh5*^*D261A*^-*Myc,* or *mrh5*^*E262A*^-*Myc*, and subjected to qRT-PCR analysis using primers specific for *cox1* and *cob1* mRNAs. Results were normalized to *act1* mRNA. Values represent the mean ± SD of at least three independent experiments. Statistically significant differences were determined by Student′s *t* test (∗, *p* < 0.05; ∗∗, *p* < 0.01). *B*, removal of the mtDNA introns could not restore Cox1 synthesis in cells harboring mutations in the DEAD-box of Mrh5. Mitochondrial translation products were labeled with [^35^S]-methionine/cysteine and analyzed by SDS/PAGE and autoradiography. *C*, deletion of mtDNA introns could not rescue the respiration growth defect caused by mutations in the DEAD-box of Mrh5. Cells were grown to log phase. Equal numbers of cells were 10-fold serial diluted (starting with OD_600_ = 2) and spotted on YES media containing glucose or glycerol.

To rule out the possibility that the mitochondrial translation defect in cells harboring mutations in the DEAD-box of Mrh5 was due to a dramatic reduction in the level of *cox1* mRNA, we constructed Δ*mrh5* mutants carrying intronless mtDNA (Δ*mrh5*[Δ*i*]) and harboring *mrh5*-*Myc*, *mrh5*^*D261A*^-*Myc* or *mrh5*^*E262A*^-*Myc* integrated into the *leu1-32* locus under the control of its endogenous promoter. Using qRT-PCR analysis, we found that removal of the introns of mtDNA in Δ*mrh5* cells harboring *mrh5*^*D261A*^-*Myc* or *mrh5*^*E262A*^-*Myc* could restore the *cox1* mRNA level to the level seen in WT[Δ*i*] cells (Fig. 6*A*). However, removal of the introns of mtDNA could not restore Cox1 synthesis (Fig. 6*B*). Consistent with these results, Δ*mrh5*[Δ*i*] cells harboring *mrh5*^*D261A*^-*Myc* or *mrh5*^*E262A*^-*Myc* could not grow on a glycerol-containing medium (Fig. 6*C*).

### The DEAD-box of Mrh5 is required for the association of Mrh5 with the mtSSU

**Figure 7:**
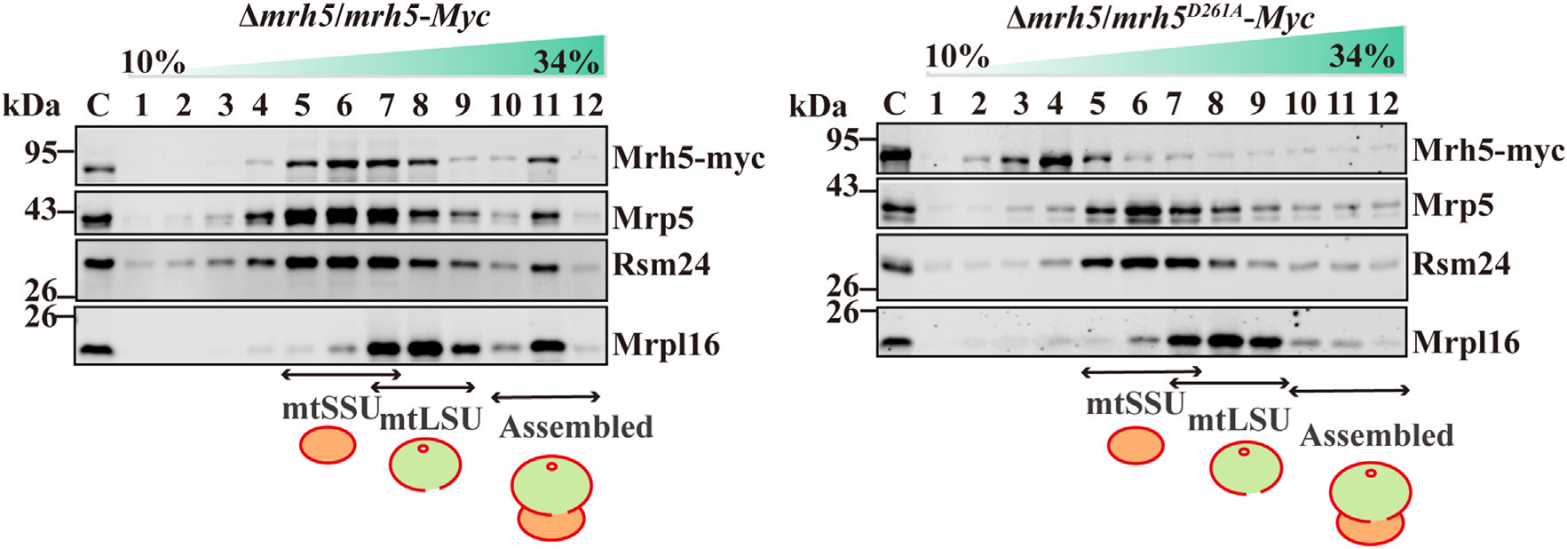
**The DEAD-box of Mrh5 is required for its association with the mtSSU.** Mutations in the DEAD-box of Mrh5 impair its association with the mtSSU. Mitochondrial extracts were prepared from Δ*mrh5* cells containing the integrated *mrh5-Myc* and *mrh5*^*D261A*^-*Myc* subjected to sucrose density centrifugation. The sucrose gradient fractions were analyzed by SDS/PAGE and immunoblotting. The positions of the mtSSU, mtLSU and mitoribosome were determined by using the mtSSU markers Mrp5 and Rsm24, and the mtLSU marker Mrpl16. *S. pombe* cells were grown to log phase and mitochondrial extracts were prepared from spheroplasts and subjected to immunoblotting for indicated proteins.

To examine whether the DEAD-box of Mrh5 is required for its association with the mtSSU, mitochondria were isolated from Δ*mrh5* cells harboring *mrh5*-*Myc*, *mrh5*^*D261A*^-*Myc* or *mrh5*^*E262A*^-*Myc* integrated into the *leu1-32* locus. Mitochondrial extracts were subjected to sucrose gradient centrifugation and distributions of Mrh5-Myc, Mrh5^D261A^-Myc, and Mrh5^E262A^-Myc in the sucrose gradient were analyzed by immunoblotting. Mutations of the DEAD-box in Mrh5-Myc resulted in the dissociation of the Mrh5 mutants with the mtSSU (Figs. 7 and S6).

### The DEAD-box of Mrh5 is required for the association of the *cox1* mRNA with the mtSSU

**Figure 8:**
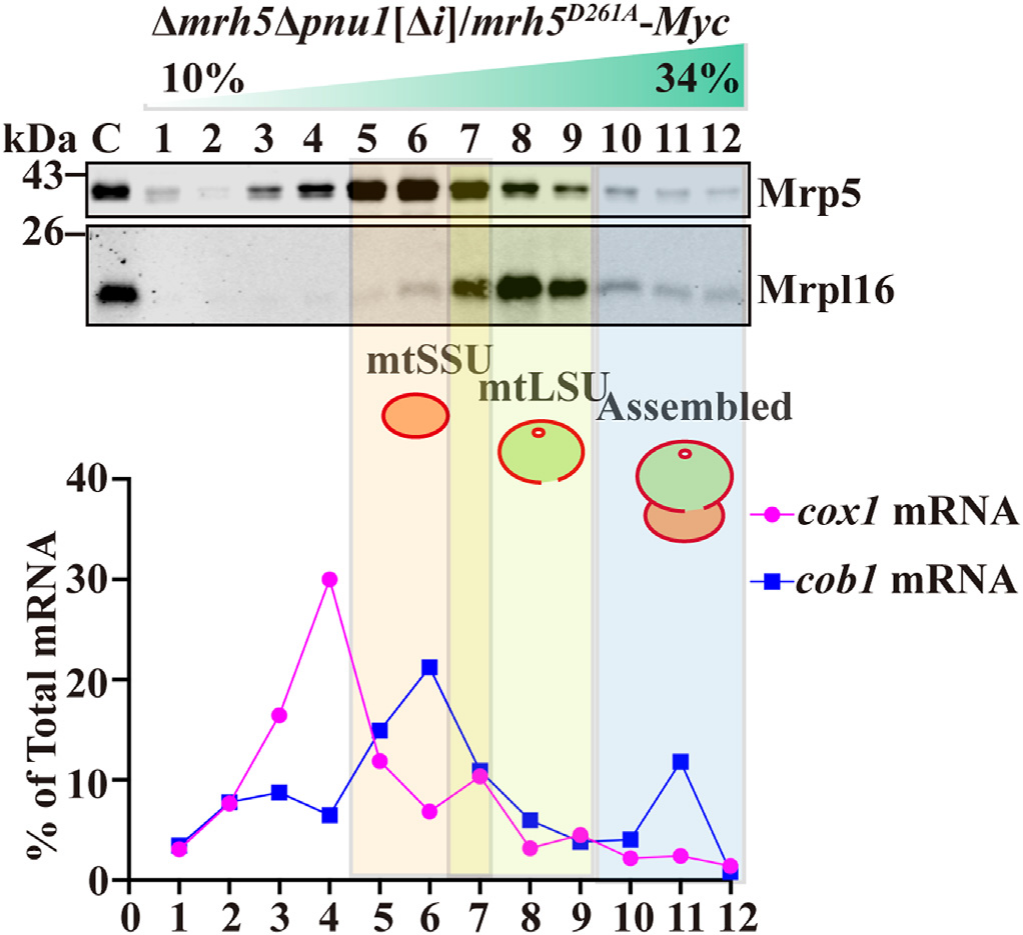
**The DEAD-box of Mrh5 is required for the association of *cox1* mRNA with the mtSSU.** The D261A mutation in the DEAD-box of Mrh5 impairs the association of *cox1* mRNA with the mtSSU. Mitochondrial extracts were prepared from Δ*mrh5*Δ*pnu1*[Δ*i*] cells containing integrated *mrh5*^*D261A*^-*Myc* and subjected to sucrose gradient centrifugation. The levels of the mtSSU marker (Mrp5) and mtLSU marker (Mrpl16) in the gradient fractions were determined by immunoblotting (*upper panels*). The levels of *cox1* and *cob1* mRNAs in the gradient fractions were analyzed by qRT-PCR (*lower panel*). The peak fractions of the mtSSU, mtLSU, and mitoribosome are indicated by transparent *orange*, *green*, and *blue* colors, respectively. The data are expressed as a percentage of total specific RNA and are representative of three independent experiments.

To address whether the DEAD-box of Mrh5 plays a role in the recruitment of *cox1* mRNA to the mtSSU, mitochondrial extracts were prepared from Δ*mrh5*Δ*pnu1*[Δ*i*] cells harboring *mrh5*^*D261A*^-*Myc* and subjected to sucrose density gradient analysis. Immunoblot analysis of factions revealed that mutation of the DEAD-box of Mrh5 abolished the association of the *cox1* mRNA with the mtSSU (Fig. 8).

### The DEAD-box of Mrh5 is required for its interaction with other Mrh5C subunits

**Figure 9:**
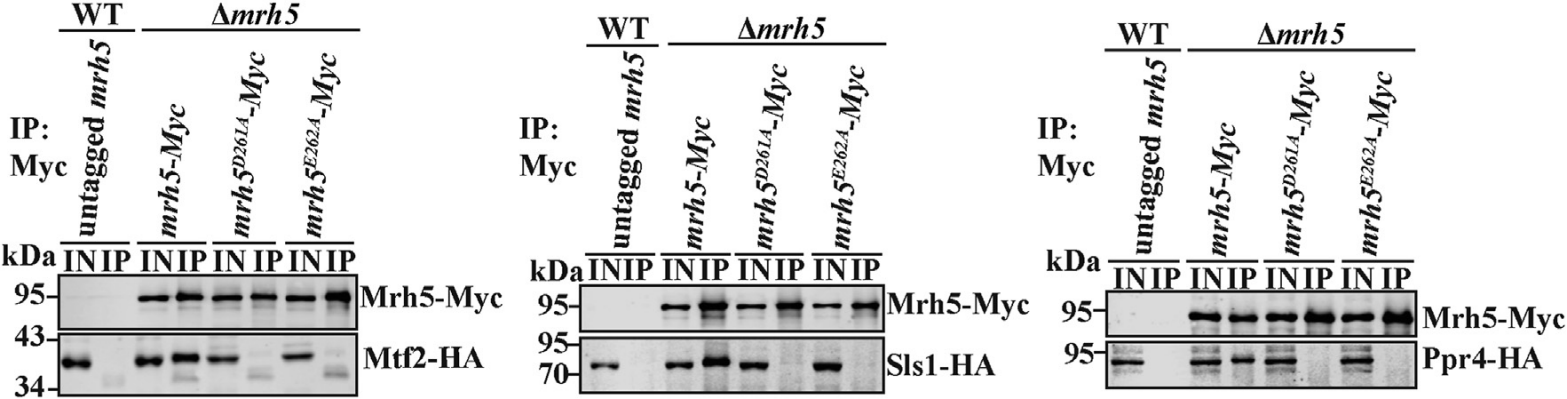
**The DEAD-box of Mrh5 is required for its association with other Mrh5C subunits.** Mitochondrial extracts prepared from WT cells expressing Mtf2-HA and untagged Mrh5, and Δ*mrh5* cells expressing Mtf2-HA and integrated *mrh5-Myc*, *mrh5*^*D261A*^*-Myc*, or *mrh5*^*E262A*^*-Myc* were subjected to anti-Myc co-immunoprecipitation (*left panels*). Mitochondrial extracts prepared from WT cells expressing Sls1-HA and untagged Mrh5, and Δ*mrh5* cells expressing Sls1-HA and integrated *mrh5-Myc*, *mrh5*^*D261A*^*-Myc*, or *mrh5*^*E262A*^*-Myc* were subjected to anti-Myc co-immunoprecipitation (*middle panels*). WT cells expressing Ppr4-HA and untagged Mrh5, and Δ*mrh5* cells expressing Ppr4-HA and integrated *mrh5-Myc*, *mrh5*^*D261A*^*-Myc*, or *mrh5*^*E262A*^*-Myc* were subjected to anti-Myc co-immunoprecipitation (*right panels*). Mitochondrial extracts (IN) and immunoprecipitates (IP) were analyzed by immunoblotting with anti-Myc and anti-HA Abs.

We determined whether mutations in the DEAD-box of Mrh5 affected its interaction with other subunits of Mrh5C. We constructed Δ*mrh5* cells bearing *mrh5*-*Myc*, *mrh5*^*D261A*^-*Myc*, or *mrh5*^*E262A*^-*Myc* and expressing HA-tagged Mtf2, Sls1, or Ppr4 from their native genomic loci. Co-immunoprecipitation experiments showed that the association of Mrh5 with other subunits of Mrh5C was abolished by mutations in the DEAD-box (Fig. 9). These results revealed that the DEAD-box is involved in the recruitment of Mrh5 into Mrh5C.

### Prediction of Ppr4 binding sites in the 5′-UTR of *cox1* mRNA

Ppr4 contains 16 putative PPR motifs. We predicted the cognate nucleotide sequence recognized by the PPR motifs of Ppr4 using the published PPR codes (41, 42) (Table S3). The predicted nucleotide sequence is 5'-(A/U)A(A/G)N(U/C/A)(A/U)(U/C)(U/C/G)(U/A)(G/U)N(A/G)(C/U/A)N(A/U)(U/C)-3', where N (G, A, T, C) represents any nucleotide that could not be precisely predicted, and the nucleotides in parentheses are optional. This predicted sequence was used to search for the Ppr4 binding sites on the *cox1* 5′-UTR using Insilicase′ Degenerate motif finder software (http://www.insilicase.com/Web/DegenerateSites.aspx). We found one sequence that fitted the degenerate pattern: AAAUAAUCUUAAUGAU, which spans from nt −141 to −126 of the *cox1* 5′-UTR (Fig. S7).

## Discussion

Mitochondrial translation is a complex process that requires both general and specific activators. Studies in budding yeast and fission yeast have shown that multiple specific activators are required for the translation of individual mtDNA-encoded mRNAs, but it remains largely unknown whether and how they interact to activate translation. Few mitochondrial translational activator complexes have been identified and characterized. In this study, we characterized Mrh5C required for the translation of *cox1* mRNA in *S. pombe*.

Our analysis provides clues to the role of two *cox1* mRNA-specific activators Mtf2 and Sls1 in Cox1 synthesis. We show that Mtf2 and Sls1 form a subcomplex required for the assembly of Mrh5C. Sls1 functions to stabilize Mtf2 within the subcomplex. The Mtf2-Sls1 subcomplex apparently acts as a scaffold to bring *cox1* mRNA-specific translational activators Mrh5 and Ppr4 into close proximity with each other. There are two possible explanations for the association between Mrh5 and Ppr4. One explanation is that because DEAD-box proteins generally cannot recognize their targets directly, and specific recognition of targets is mainly provided by the binding partners (43), it is likely that Ppr4 is required for the specific binding of Mrh5 to *cox1* mRNA. Another possible explanation is that the association between Mrh5 and Ppr4 is required to coordinate the actions of these proteins.

Our study also provides insights into the role of Mrh5C in the activation of *cox1* mRNA translation. We show that the whole Mrh5C complex, but not the individual subunits, was associated with the mtSSU. In addition, we show that Mrh5C subunits play a role in *cox1* mRNA association with the mtSSU and mitoribosome only when they are present as a Mrh5C complex. Together with our previous studies demonstrating that Mrh5C is specifically associated with *cox1* mRNA (37), our findings support a role for Mrh5C in the recruitment of *cox1* mRNA to the mtSSU during translation initiation. We speculate that Ppr4 may play a role in the recruitment of *cox1* mRNA to the mtSSU through specific binding to the *cox1* mRNA 5′-UTR, while Mrh5 may cooperate with Ppr4 to facilitate mitoribosome loading on *cox1* mRNA via unwinding of the *cox1* mRNA secondary structure in the *cox1* mRNA 5′-UTR. Using computational analysis, we have identified a potential binding site of Ppr4 on the *cox1* mRNA 5′-UTR. Consistent with the above speculation, we found that the DEAD-box of Mrh5 plays an important role in *cox1* mRNA translation. However, it remains to be determined the detailed mechanism by which the entire Mrh5C complex functions as a whole to recognize and bind *cox1* mRNA.

Unexpectedly, the amino acid residues predicted to be important for the ATPase activity of Mrh5 are also involved in protein-protein interactions that allow the recruitment of Mrh5 to Mrh5C. This observation is not without precedent. It has been reported that a mutation in the DEAD-box (Glu183 to Gln183) of the RNA helicase eIF4AII impairs the interaction of eIF4AII with its binding partner (44). It is presently unclear how the mutations that can potentially abolish Mrh5′s ATPase and/or helicase activity also abolish its ability to interact with other subunits of Mrh5C. Because the DEAD-box is involved in the interaction with Mg^2+^ required for ATP binding (45), the most likely explanation is that mutation of the DEAD-box of Mrh5 causes conformational changes around the sites in the proteins involved in interaction with other subunits of Mrh5C, leading to the dissociation of Mrh5 from the complex.

As suggested by the finding that the *S. cerevisiae* DEAD-box helicase Mss116 and PPR protein Pet309 interact with each other and function together to activate *cox1* mRNA translation, a complex similar to Mrh5C may exist in *S. cerevisiae* (8). However, the formation and composition of the *S. cerevisiae cox1* mRNA-specific translational activator complex remain to be fully characterized. Apparently, there are differences between the *S. pombe* and *S. cerevisiae cox1*-specific translational activator complexes. Firstly, Mss116 and Pet309 do not exhibit significant sequence similarity to Mrh5 and Ppr4, respectively. Secondly, unlike the situation in *S. pombe*, Mss116 is required for the stability of Pet309. Thirdly, *S. cerevisiae* Sls1 and Mtf2, which have sequence similarity to *S. pombe* Sls1 and Mtf2, respectively, are required for global mitochondrial translation (18). It remains unclear whether the mechanisms of activation of *cox1* mRNA translation in *S. pombe* and *S. cerevisiae* derive from convergent evolution or a common ancestral mechanism that controls the translation of *cox1* mRNA.

It is interesting to note that DEAD-box proteins and PPR proteins have been shown to form complexes, and these complexes function in organellar RNA metabolism. In *S. cerevisiae*, Pet309 and Mss116 cooperate in the translation of the *cox1* mRNA (8). In humans, DEAD-box protein DHX30 and PPR protein PTCD3 associate with each other and play a role in mtDNA transcription (46). In maize, mitochondrial RNA splicing complexes containing DEAD-box RNA helicases and PPR proteins have been identified (47, 48). DEAD-box RNA helicase PMH2-5140 associates with PPR protein EMP603 and is required for the splicing of the intron in the mitochondrial NADH dehydrogenase subunit 1 gene (*nad1*) (48). However, the biological significance of the association between DEAD-box proteins and PPR proteins remains to be determined.

## Experimental procedures

### Strains, media, and genetic methods

*S. pombe* strains used in this article are listed in Table S1. *S. pombe* deletion strains were constructed by homologous recombination as described (49). Because *S. pombe* cells defective in mitochondrial respiration failed to grow in minimal medium (21), antibiotic resistance markers (*kanMX6*, *hphMX6*, or *natMX6*) were used as a selectable marker for gene deletion, which allows selection of transformants on rich medium. The deletion cassettes containing antibiotic resistance markers were generated by overlap PCR and transformed into appropriate *S. pombe* strains by the lithium acetate method (50). All deletion constructs were verified by PCR.

For C-terminal Myc tagging of endogenous Mrh5, the 5′ and 3′ flanking sequences of *mrh5* were amplified by PCR using the *S. pombe* genomic DNA as a template and cloned into the Sal I/Sma I sites and Sac I/Sac II sites of pFA6A-13Myc-kanMX6 (49), respectively. All other tagging cassettes were generated by overlap PCR. The *CBP*-*leu1* cassette for C-terminal CBP tagging of endogenous Ppr4 was generated as follows: the 5′ flanking sequence immediately upstream of the *ppr4* stop codon and the 3′ flanking sequence immediately downstream of the *ppr4* stop codon were amplified by PCR using the *S. pombe* genomic DNA as template. A third DNA fragment containing the CBP tag coding sequence, the *S. cerevisiae* alcohol dehydrogenase 1 gene (*ADH1*) terminator, and the *leu1* gene was obtained by PCR using plasmid pFA6a-3HA-leu1 (21) as template and PCR primers containing the nucleotide sequence coding for the CBP tag. These three PCR fragments were fused by PCR and the resulting PCR product was transformed into appropriate *S. pombe* strains. pFA6a-2FLAG-phpMX6 (21) was used for the addition of the FLAG tag to the C-terminus of Sls1. pK18-3HA-natMX6 (see below description) was used for generating C-terminally HA-tagged Mtf2, Sls1, Ppr4 and Mrp51. pFA6a-3HA-leu1 (21) was also used for adding the HA tag to the C-terminus of Mtf2.

To generate strains harboring integrated *mrh5*-*Myc*, *mrh5*^*D261A*^-*Myc*, or *mrh*5^*E262A*^-*Myc* under the control of the *mrh5* promoter into the *leu1-32* locus, a DNA fragment containing the promoter sequence of *mrh5* and the coding sequence of *mrh5* was amplified from *S. pombe* genomic DNA and cloned into the BamH I/Smal I sites of pZL1 (20), generating plasmid pWY1. Mutations in the DEAD-box of Mrh5 were generated by PCR-based site-directed mutagenesis using the Mut Express II Fast Mutagenesis kit V2 (Vazyme) with pWY1 as a template. PCR products were digested with Dpn I, and transformed into DH5α. Plasmid DNA was isolated and mutations were confirmed by DNA sequencing. Plasmids expressing Mrh5-Myc, Mrh5^D261A^-Myc, and Mrh5^E262A^-Myc were digested with Nru I and transformed into the Δ*mrh5* strain. Transformants were selected on YES medium containing 100 μg/ml hygromycin B. Expression of Mrh5-Myc, Mrh5^D261A^-Myc, or Mrh5^E262A^-Myc was confirmed by immunoblotting.

*S. pombe* cells are routinely grown at 30 °C in rich media containing 0.5% yeast extract and 3% glucose supplemented with 250 mg/L adenine, 250 mg/L uracil, 250 mg/L leucine and 250 mg/L histidine (YES) for fermentative growth or 3% glycerol and 0.1% glucose for respiratory growth (50). Cells were grown in a synthetic minimal medium (EMM) with the appropriate auxotrophic supplements for the isolation of Leu^+^ transformants. Standard media and protocols for the genetic manipulation of fission yeast were used as described previously (50).

### Plasmid construction

The *3HA-natMX6* cassette was constructed by PCR amplification of genomic DNA from the *S. pombe* strain NG60 (51) using PCR primers containing the nucleotide sequence coding for the HA tag, and cloned into Xba I/Sal I sites of pK18mobsacB (52), generating plasmid pK18-3HA-natMX6.

### Quantitative real-time PCR (qRT-PCR)

*S. pombe* cells were grown to the exponential phase in YES medium. Cells were lysed by VinoTaste Pro (Novozymes). Total RNA was isolated using the RNeasy Mini kit (Qiagen). Contaminating DNA was removed by treatment with RNase-free DNase I (Qiagen). RNA was reverse transcribed using HiScript III RT SuperMix for qPCR (+gDNA wiper) (Vazyme). qRT-PCR was carried out with Taq Pro Universal SYBR qPCR Master Mix (Vazyme) with primers specific for mtDNA (Table S2). Relative-fold changes in mt-mRNA abundance in Δ*mrh5* cells with integrated empty vector, or the same vector carrying the WT or mutant alleles of *mrh5* under control of its endogenous promoter relative to WT cells were calculated using the 2-^ΔΔCt^ method after normalization to *act1* mRNA. Statistical significance was calculated using Prism software (GraphPad Software). Error bars represent the standard deviations of three independent experiments (39).

### Preparation of mitochondrial extracts

Mitochondrial extracts were prepared as described except that cells were lysed by VinoTaste Pro (Novozymes) (53). Briefly, *S. pombe* cells were lysed in buffer containing 40 mM HEPES-KOH, pH 6.5, 1.4 M sorbitol, 1.5 mM MgCl_2_, and 50 mg/ml of VinoTaste Pro at 30 °C on a rotary shaker at 200 rpm for 2 to 3 h. The spheroplasts were harvested by centrifugation at 2057*g* for 5 min, resuspended in a buffer containing 10 mM Tris-HCl, pH 7.4, 600 mM sorbitol and 1 mM PMSF and disrupted using a Dounce homogenizer (Sigma). Unbroken spheroplasts, cell debris and nuclei were removed by centrifugation at 2057*g* once and 3214*g* twice. The mitochondria were pelleted by centrifugation at 15,557*g* for 15 min followed by washing once with a buffer containing 20 mM HEPES-KOH, pH 7.4, and 600 mM sorbitol, and frozen at −80 °C until use. For immunoblotting, mitochondria were solubilized in the SDS sample buffer and analyzed by immunoblotting. For immunoprecipitation, the mitochondria were lysed in buffer containing 20 mM Tris-HCl, pH 7.4, 150 mM NaCl, 10% glycerol, 0.1 M PMSF, 1% Igepal CA-630, and cOmplete EDTA-free protease inhibitor cocktail tablets (Roche Diagnostics) for 20 min on ice. For sucrose gradient analysis, mitochondrial extracts were prepared by lysing mitochondria (∼2 mg) in lysis buffer containing 20 mM HEPES-KOH, pH 7.4, 100 mM KCl, 10 mM MgCl_2_, 0.5 mM PMSF, EDTA-free complete protease inhibitor (Roche) and 1% dodecyl maltoside (DDM). After 30 min on ice, the suspension was centrifuged for min at 15,700*g* and the supernatant was collected.

### Labeling of mitochondrial translation products

*In vivo* labeling of mitochondrial translation products was carried out as essential described previously (21). Cells were grown in YES media containing 5% raffinose and 0.1% glucose to early-log phase. For [^35^S]-methionine labeling, cells were incubated for 15 min in a buffer consisting of 40 mM potassium phosphate buffer, pH 6.0, 5% raffinose, 0.1% glucose, and anisomycin (1 mg/ml), and then labeled with 176 μCi/ml [^35^S]-methionine/cysteine (PerkinElmer Life Science) for 3 h. Reactions were stopped by the addition of cell solubilization buffer (1.8 M NaOH, 1 M *β*-mercaptoethanol, and 10 mM PMSF). Products of the reactions were precipitated by TCA, separated by SDS/PAGE, and visualized by autoradiography.

### Sucrose gradient analysis

Sucrose gradient analysis was performed essentially as described previously (20). Briefly, mitochondrial extracts were layered onto a 10 to 34% sucrose gradient, and ultracentrifuged at 260,800*g* for 3 h at 4 °C in a Beckman SW60Ti rotor. 12 fractions (350 μl) were collected from the bottom to the top of the gradient. For analysis of the association of Mrh5C with the mitoribosome, proteins were precipitated in 25% trichloroacetic acid (TCA), collected by centrifugation, washed with acetone, and analyzed by immunoblotting. For analysis of the association of mt*-*mRNA with the mitoribosome, each fraction was divided into one-third and two-third portions. Two-thirds of the sample was used for total RNA extraction (54), followed by qRT-PCR quantification of *cox1* and *cob1* mRNAs, while the remainder was used for immunoblotting of the mitoribosome.

### Immunoblotting analysis

Proteins were separated using SDS/PAGE and transferred to nitrocellulose membranes (GE Healthcare Life Science). Anti-peptide Abs against Cox1, Cox2, Cox3, Cob1, and Atp6 were prepared as described previously (21). Anti-peptide Abs against Mrp5, Rsm24, Mrpl16, and Mrpl40 were generated as described (55). Other primary Abs used are anti-HA (Cat. No. T0008, Affinity Biosciences), anti-FLAG (Cat. No. T0003, Affinity Biosciences), anti-CBP (Cat. No. A00635, Genscript Biotech Corporation), anti-Myc (Cat. No. T0001, Affinity Biosciences), and anti-HSPD1 (Cat. No. D120788, Sangon Biotech) for detection of Mcp60. The secondary Abs were IRDye 800CW goat anti-rabbit Ab (Cat. No. 926-32211, LI-COR Biosciences) and IRDye 800CW goat anti-mouse Ab (Cat. No. 926-32210, LI-COR Biosciences). Bands were detected using an Odyssey near-infrared fluorescence scanner (LI-COR Biosciences), and quantified using Image Studio Lite software (LI-COR Biosciences).

## Data availability

All the data are contained within the article and supporting information. Data can be made available upon request of the lead contact.

## Supporting information

This article contains supporting information (56, 57).

## Conflict of interest

The authors declare that they have no conflict of interest with the contents of this article.
