# Supplementary material for: Sls1 and Mtf2 mediate the assembly of the Mrh5C complex required for activation of cox1 mRNA translation
Source: J Biol Chem. 2024 Mar 16;300(4):107176. doi: 10.1016/j.jbc.2024.107176 (PMC11015131; doi:10.1016/j.jbc.2024.107176)
Supplement: Supplementary Figure S1–S7 and Tables S1–S3 [file mmc1.docx]

**Supporting information**

Sls1 and Mtf2 mediate the assembly of the Mrh5C complex required for activation of *cox1* mRNA translation

Yirong Wang, Ting Jin and Ying Huang*

Jiangsu Key Laboratory for Microbes and Functional Genomics, Nanjing Normal University, 1 Wenyuan Road, Nanjing 210023, China

*Corresponding author

Ying Huang, Ph.D.

Jiangsu Key Laboratory for Microbes and Genomics

Nanjing Normal University

1 Wenyuan Road, Nanjing 210023

China

Tel: 01186-25-85891263

Fax: 01186-25-85891263

E-mail: [yhuang@njnu.edu.cn](mailto:yhuang@njnu.edu.cn)

Contents:

Supporting information Table S1

Supporting information Table S2

Supporting information Table S3

Supporting information Figure S1

Supporting information Figure S2

Supporting information Figure S3

Supporting information Figure S4

Supporting information Figure S5

Supporting information Figure S6

Supporting information Figure S7

**Table S1** *S. pombe* strains used in this study

| **Strain** | **Genotype** | **Source** |
| --- | --- | --- |
| yHL6381 | *h^+^ leu1-32 his3-D1 ura4-D18 ade6-M210* | H. Levin |
| P3 | *h^+^ ade7-50 rho^+^* (no mitochondrial introns) | N. Bonnefoy |
| yWY1 | *h^+^ leu1-32 his3-D1 ura4-D18 ade6-M210* *mtf2*::[*mtf2*-*3HA*-*leu1^+^*] | This paper |
| yWY2 | *h^+^ leu1-32 his3-D1 ura4-D18 ade6-M210* *mrp51*::[*mrp51*-*3HA*-*natMX6*] | This paper |
| yWY3 | *h^+^ leu1-32 his3-D1 ura4-D18 ade6-M210* *mtf2*::[*mtf2*-*3HA*-*leu1^+^*] *sls1*::[*sls1*-*2FLAG*-*hphMX6*] | This paper |
| yWY4 | *h^+^ leu1-32 his3-D1 ura4-D18 ade6-M210* *mrh5*::[*mrh5*-*13Myc*-*kanMX6*] *ppr4*::[*ppr4*-*CBP*-*leu1^+^*] *mtf2*::[*mtf2*-*3HA*-*natMX6*] | This paper |
| yWY5 | *h^+^ leu1-32 his3-D1 ura4-D18 ade6-M210* *ppr4*::[*ppr4*-*CBP*-*leu1^+^*] *sls1*::[*sls1*-*2FLAG* -*hphMX6*] *mtf2*::[*mtf2*-*3HA*-*natMX6*] | This paper |
| yWY6 | *h^+^ leu1-32 his3-D1 ura4-D18 ade6-M210* *mrh5*::[*mrh5*-*13Myc*-*kanMX6*] *ppr4*::[*ppr4*-*CBP*-*leu1^+^*] *sls1*::[*sls1*-*2FLAG*-*hphMX6*] *mtf2*::[*mtf2*-*3HA*-*natMX6*] | This paper |
| yWY7 | *h^+^ leu1-32 his3-D1 ura4-D18 ade6-M210* Δ*sls1*::*kanMX6 mrp51*::[*mrp51*-*3HA*-*natMX6*] | This paper |
| yWY8 | *h^+^ leu1-32 his3-D1 ura4-D18 ade6-M210* Δ*mrh5*::*kanMX6 mrp51*::[*mrp51*-*3HA*-*natMX6*] | This paper |
| yWY9 | *h^+^ leu1-32 his3-D1 ura4-D18 ade6-M210* Δ*ppr4*::*kanMX6*  *mrp51*::[*mrp51*-*3HA*-*natMX6*] | This paper |
| yWY10 | *h^+^ leu1-32 his3-D1 ura4-D18 ade6-M210* Δ*mtf2*::*kanMX6 mrp51*::[*mrp51*-*3HA*-*natMX6*] | This paper |
| yWY11 | *h^+^ leu1-32 his3-D1 ura4-D18 ade6-M210* Δ*mrh5*::*kanMX6* | This paper |
| yWY12 | *h^+^ leu1-32 his3-D1 ura4-D18 ade6-M210* Δ*mrh5*::*kanMX6 mrh5*::[*mrh5*-*13Myc*-*hphMX6*] | This paper |
| yWY13 | *h^+^ leu1-32 his3-D1 ura4-D18 ade6-M210* Δ*mrh5*::*kanMX6 mrh5*::[*mrh5^D261A^*-*13Myc*-*hphMX6*] | This paper |
| yWY14 | *h^+^ leu1-32 his3-D1 ura4-D18 ade6-M210* Δ*mrh5*::*kanMX6 mrh5*::[*mrh5^E262A^*-*13Myc*-*hphMX6*] | This paper |
| yWY15 | *h^+^ leu1-32 his3-D1 ura4-D18 ade6-M210* Δ*mrh5*::*kanMX6 mrh5*::[*mrh5*-*13Myc*-*hphMX6*] *mtf2*::[*mtf2*-*3HA*-*natMX6*] | This paper |
| yWY16 | *h^+^ leu1-32 his3-D1 ura4-D18 ade6-M210* Δ*mrh5*::*kanMX6 mrh5*::[*mrh5*-*13Myc*-*hphMX6*] *sls1*::[*sls1*-*3HA*-*natMX6*] | This paper |
| yWY17 | *h^+^ leu1-32 his3-D1 ura4-D18 ade6-M210* Δ*mrh5*::*kanMX6 mrh5*::[*mrh5*-*13Myc*-*hphMX6*] *ppr4*::[*ppr4*-*3HA*-*natMX6*] | This paper |
| yWY18 | *h^+^ leu1-32 his3-D1 ura4-D18 ade6-M210* Δ*mrh5*::*kanMX6 mrh5*::[*mrh5^D261A^*-*13Myc*-*hphMX6*] *mtf2*::[*mtf2*-*3HA*-*natMX6*] | This paper |
| yWY19 | *h^+^ leu1-32 his3-D1 ura4-D18 ade6-M210* Δ*mrh5*::*kanMX6 mrh5*::[*mrh5^D261A^*-*13Myc*-*hphMX6*] *sls1*::[*sls1*-*3HA*-*natMX6*] | This paper |
| yWY20 | *h^+^ leu1-32 his3-D1 ura4-D18 ade6-M210* Δ*mrh5*::*kanMX6 mrh5*::[*mrh5^D261A^*-*13Myc*-*hphMX6*] *ppr4*::[*ppr4*-*3HA*-*natMX6*] | This paper |
| yWY21 | *h^+^ leu1-32 his3-D1 ura4-D18 ade6-M210* Δ*mrh5*::*kanMX6 mrh5*::[*mrh5^E262A^*-*13Myc*-*hphMX6*] *mtf2*::[*mtf2*-*3HA*-*natMX6*] | This paper |
| yWY22 | *h^+^ leu1-32 his3-D1 ura4-D18 ade6-M210* Δ*mrh5*::*kanMX6 mrh5*::[*mrh5^E262A^*-*13Myc*-*hphMX6*] *sls1*::[*sls1*-*3HA*-*natMX6*] | This paper |
| yWY23 | *h^+^ leu1-32 his3-D1 ura4-D18 ade6-M210* Δ*mrh5*::*kanMX6 mrh5*::[*mrh5^E262A^*-*13Myc*-*hphMX6*] *ppr4*::[*ppr4*-*3HA*-*natMX6*] | This paper |
| yWY24 | *h^+^ leu1-32 his3-D1 ura4-D18 ade6-M210* Δ*mrh5*::*kanMX6 ppr4*::[*ppr4-CBP-leu1^+^*] *mtf2*::[*mtf2*-*3HA*-*natMX6*] *sls1*::[*sls1*-*2FLAG*-*hphMX6*] | This paper |
| yWY25 | *h^+^ leu1-32 his3-D1 ura4-D18 ade6-M210* Δ*ppr4*::*natMX6 mrh5*::[*mrh5*-*13Myc*-*kanMX6*] *mtf2*::[*mtf2*-*3HA*-*leu^+^*] *sls1*::[*sls1*-*2FLAG*-*hphMX6*] | This paper |
| yWY26 | *h^+^ leu1-32 his3-D1 ura4-D18 ade6-M210* Δ*mtf2*::*natMX6 mrh5*::[*mrh5*-*13Myc*-*kanMX6*] *ppr4*::[*ppr4-CBP-leu1^+^*] *sls1*::[*sls1*-*2FLAG*-*hphMX6*] | This paper |
| yWY27 | *h^+^ leu1-32 his3-D1 ura4-D18 ade6-M210* Δ*mrh5*::*kanMX6* Δ*ppr4*::*natMX6 mtf2*::[*mtf2*-*3HA*-*leu1^+^*] *sls1*::[*sls1*-*2FLAG*-*hphMX6*] | This paper |
| yWY28 | *h^+^ ade7-50 rho^+^* (no mitochondrial introns) Δ*mrh5*::*kanMX6* | This paper |
| yWY29 | *h^+^ ade7-50 rho^+^* (no mitochondrial introns) Δ*mrh5*::*kanMX6 mrh5*::[*mrh5*-*13Myc*-*hphMX6*] | This paper |
| yWY30 | *h^+^ ade7-50 rho^+^* (no mitochondrial introns) Δ*mrh5*::*kanMX6 mrh5*::[*mrh5^D261A^*-*13Myc*-*hphMX6*] | This paper |
| yWY31 | *h^+^ ade7-50 rho^+^* (no mitochondrial introns) Δ*mrh5*::*kanMX6 mrh5*::[*mrh5^E262A^*-*13Myc*-*hphMX6*] | This paper |
| yWY32 | *h^+^ ade7-50 rho^+^* (no mitochondrial introns) Δ*pnu1*::*kanMX6* | This paper |
| yWY33 | *h^+^ ade7-50 rho^+^* (no mitochondrial introns) Δ*ppr4*::*hphMX6* Δ*pnu1*::*kanMX6* | This paper |
| yWY34 | *h^+^ ade7-50 rho^+^* (no mitochondrial introns) Δ*mrh5*::*kanMX6* Δ*pnu1*::*hphMX6* | This paper |
| yWY35 | *h^+^ ade7-50 rho^+^* (no mitochondrial introns) Δ*mtf2*::*natMX6* Δ*pnu1*::*kanMX6* | This paper |
| yWY36 | *h^+^ ade7-50 rho^+^* (no mitochondrial introns) Δ*mrh5*::*kanMX6* Δ*pnu1*::*natMX6 mrh5*::[*mrh5^D261A^*-*13Myc*-*hphMX6*] | This paper |

**Table S2** Primers are used for site-directed mutagenesis and qRT-PCR

| **Name** | **Primer sequence (5′ to 3′)** |
| --- | --- |
| **Primer sequences for site-directed mutagenesis** | |
| *mrh5D261A-*f | AATTGTTGCCGAATCAGATTTAGTAATTCCCGA |
| *mrh5D261A-*r | CTGATTCGGCAACAATTAATAATTGAAGTTGTGAAGTAA |
| *mrh5E262A-*f | TGTTGACGCATCAGATTTAGTAATTCCCGATCACG |
| *mrh5E262A-*r | AATCTGATGCGTCAACAATTAATAATTGAAGTTGTG |
| **Primer sequences for qRT-PCR analysis** | |
| *actin-*f | AAGGCTAGCTCTGCATTCGTCTAT |
| *actin-*r | TCCGCTCTTAACATCTCATGAGG |
| *cox1-*f | TGGACGGTATATCCACCACT |
| *cox1-*r | GTCGCTATTAAATTTACTGATCC |
| *cox2-*f | AAGTGGTGATGTTATCCATAGTTGG |
| *cox2*-r | AGATACACCTTGAACAACAATAGGC |
| *cox3-*f | CCACCAGTAGGAATAGCAGATAAAA |
| *cox3-r* | TGAGCATAAGTTAAACTAGCACCAG |
| *cob1-*f | GCCTTTTGTTATTGCTGCTTTA |
| *cob1-*r | GTTATCAAAT CTTTTATCAG ATAAT |
| *atp6-*f | TACCTTCTGGTACTCCTACTCC |
| *atp6-*r | TAGCACCTAATCGAATACCTAAACTT |
| *atp8-*f | ATGCCACAATTAGTACCATTCT |
| *atp8-*r | AAAGAACTTATAATAGATCTTGAG |
| *atp9-*f | GGTGCTGGTGTTGGTATTGGA |
| *atp9-*r | ACCTGTAGCTTCTGTTAAGGCG |
| *var1-*f | AGAGCTCTTCCTATTTCAACTCCTT |
| *var1-*r | ACCTTTCCATCCTTTTGGTACA |
| *rns-*f | GAAGGAGGAATTGCGAGTAATCAC |
| *rns-*r | CGACTTAACACTAATTGCACAACACC |
| *rnl-*f | GTAGCACGGTAGTAAAGCCAAATTG |
| *rnl-*r | TAAGGATTTGTACATCCTAAGGATGTCC |

**Table S3** Prediction of the binding sites on the *cox1* mRNA 5’-UTR of Ppr4

| Ppr4 PPR motif | Amino acid position in Ppr4 | | Reported Amino acid combination | | Probability of nucleotide binding | | | | Predicted nucleotide in the *cox1* 5 ́-UTR | Sequence in the *cox1* mRNA 5 ́-UTR |
| --- | --- | --- | --- | --- | --- | --- | --- | --- | --- | --- |
|  | 5 | 35 | 5 | 35 | A | C | G | U |  | -141 |
| 1 | S | L | S | * | 0.38 | 0.13 | 0.20 | 0.29 | A>U | A |
| 2 | S | N | S | N | 0.63 | 0.08 | 0.05 | 0.24 | A | A |
| 3 | T | S | T | S | 0.31 | 0.24 | 0.17 | 0.28 | A>G | A |
| 4 | R | A | Not reported | |  | | | | A, C, G, U | U |
| 5 | N | P | N | * | 0.11 | 0.33 | 0.10 | 0.45 | U>C>A | A |
| 6 | S | Y | S | * | 0.38 | 0.13 | 0.20 | 0.29 | A>U | A |
| 7 | L | R | L | * | 0.20 | 0.30 | 0.03 | 0.47 | U>C | U |
| 8 | N | D | N | D | 0.11 | 0.18 | 0.10 | 0.61 | U>C>G | C |
| 9 | V | S | V | S | 0.31 | 0.29 | 0.06 | 0.35 | U>A | U |
| 10 | A | D | A | D | 0.16 | 0.10 | 0.53 | 0.21 | G>U | U |
| 11 | R | Q | Not reported | |  | | | | A, C, G, U | A |
| 12 | T | Y | T | * | 0.45 | 0.09 | 0.31 | 0.15 | A>G | A |
| 13 | N | S | N | S | 0.11 | 0.47 | 0.07 | 0.36 | C>U>A | U |
| 14 | D | P | Not reported | |  | | | | A, C, G, U | G |
| 15 | S | I | S | * | 0.38 | 0.13 | 0.20 | 0.29 | A>U | A |
| 16 | I | F | I | * | 0.15 | 0.29 | 0.10 | 0.45 | U>C | U |

Figure S1


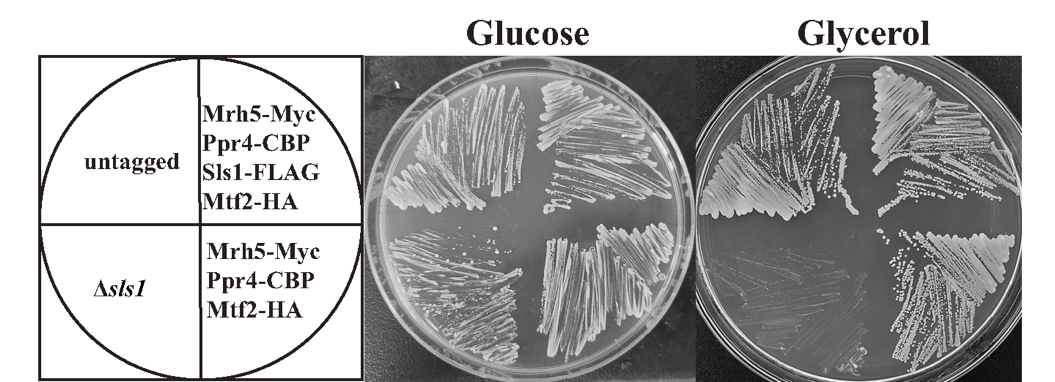


**Fig. S1.** **Tagging of Mrh5C subunits have no effect on the function of Mrh5C.** WT cells expressing untagged Mrh5C subunits (positive control), Δ*sls1* cells (negative control), WT cells expressing Mrh5-Myc, Ppr4-CBP, Sls1-FLAG and Mtf2-HA, or Mrh5-Myc, Ppr4-CBP and Mtf2-HA were plated on rich media containing glucose or glycerol as the major carbon source.

Figure S2


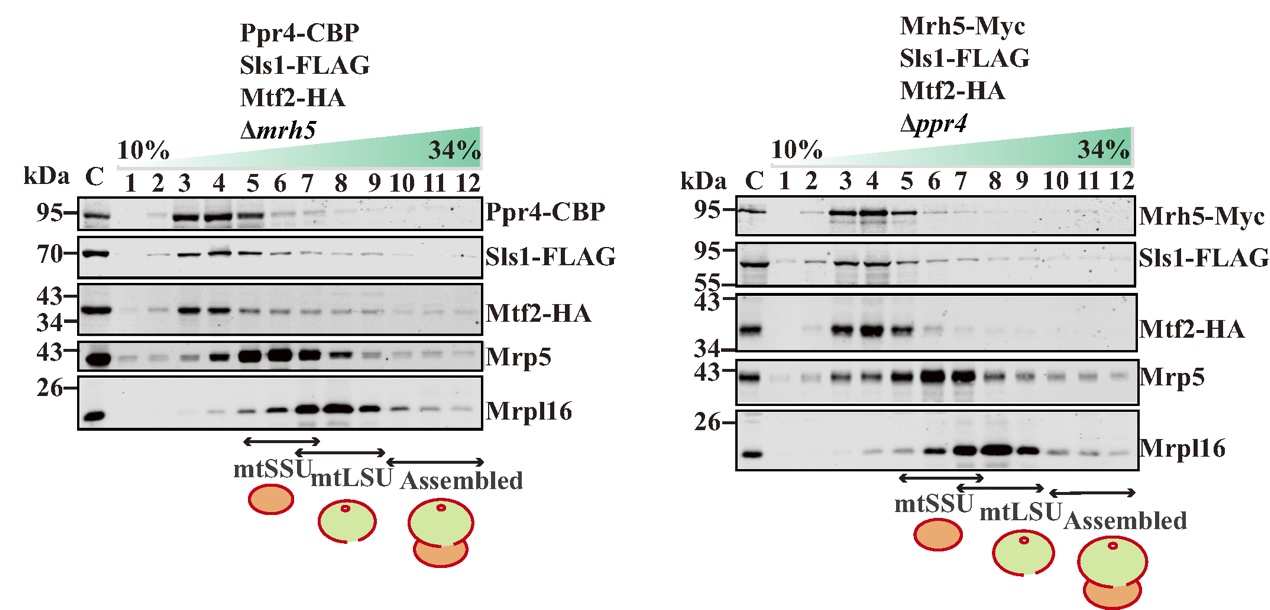


**Fig. S2. Loss of *mrh5* or *ppr4* impairs Mrh5C association with mtSSU.** Δ*mrh5* cells expressing Ppr4-CBP, Sls1-FLAG and Mtf2-HA (left panels) and Δ*ppr4* cells expressing Mrh5-Myc, Sls1-FLAG and Mtf2-HA (right panels) were subjected to 10%-34% sucrose density gradient centrifugation. Gradient fractions (1, top; 12, bottom), were analyzed by immunoblotting with specific anti-tag Abs. The mtSSU protein Mrp5 and the mtLSU protein Mrpl16 indicate the distribution of mitoribosomal complexes. C, total mitochondrial proteins.

Figure S3


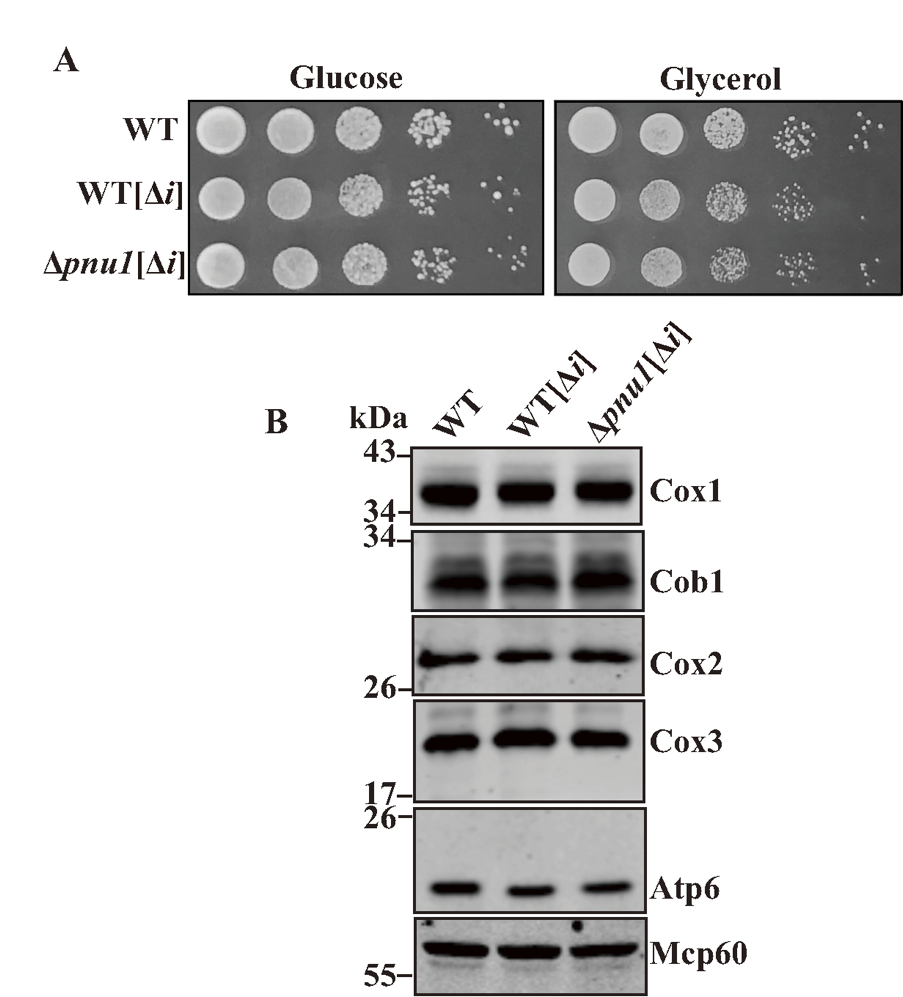


**Fig. S3.** **Deletion of *pnu1* does not impair respiratory growth.** (A) WT cells, WT cells devoid of mtDNA [WT(Δ*i*)] and Δ*pnu1* cells devoid of mtDNA (Δ*pnu1*[Δ*i*]) were grown to stationary phase. Equal numbers of cells were 10-fold serial diluted and spotted on rich media containing glucose or glycerol. (B) Mitochondrial extracts prepared from WT cells, WT[Δ*i*] and Δ*pnu1*[Δ*i*] cells were subjected to immunoblotting with Abs specific to the mtDNA-encoded proteins. Mcp60 serves as a loading control.

Figure S4


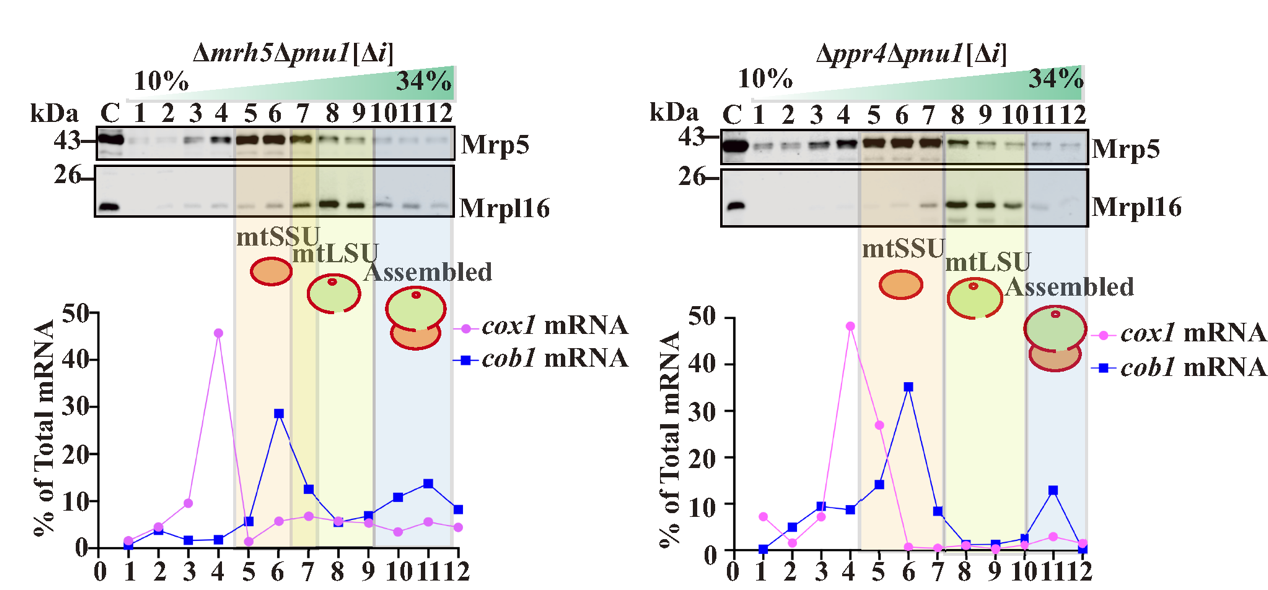


**Fig. S4. Deletion of *mrh5* or *ppr4* abolishes the association of *cox1* mRNA with mtSSU.** Mitochondrial extracts prepared from mtDNA intronless Δ*mrh5*Δ*pnu1* cells (Δ*mrh5*Δ*pnu1*[Δ*i*]) (left panels) and mtDNA intronless Δ*ppr4*Δ*pnu1* cells (Δ*ppr4*Δ*pnu1*[Δ*i*]) cells (right panels) were used for sucrose gradient centrifugation. The positions of mitoribosomal complexes on sucrose gradients were determined by immunoblotting using anti-Mrp5 and anti-Mrpl16 Abs (upper panels). The distribution of *cox1* and *cob1* mRNAs on sucrose gradients was determined by qRT-PCR (lower panels). The peak fractions of the mtSSU, mtLSU, and assembled mitoribosome are marked by transparent orange, green and blue colors, respectively. The data are expressed as percentage of total specific RNA and are representative of three independent experiments.

Figure S5


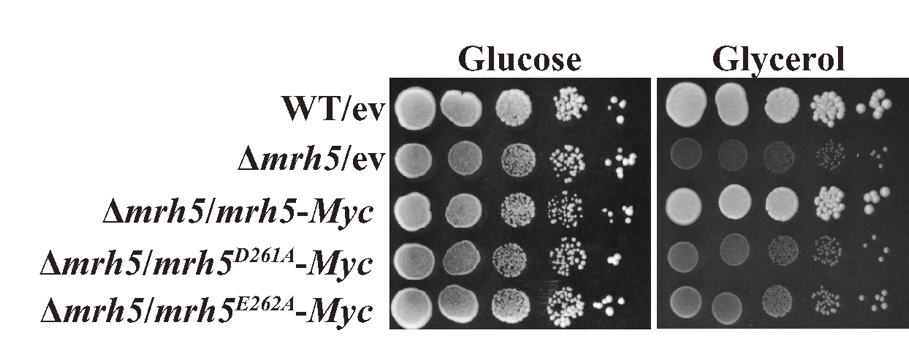


**Fig. S5. Mutations in DEAD-box of Mrh5 impair respiratory growth.** WT cells bearing the empty vector (ev), Δ*mrh5* cells harboring integrated ev, *mrh5*-*Myc*, *mrh5^D261A^*-*Myc* or *mrh5^E262A^*-*Myc* were grown to stationary phase. Equal numbers of cells were 10-fold serial diluted and spotted on rich media containing glucose or glycerol.

Figure S6


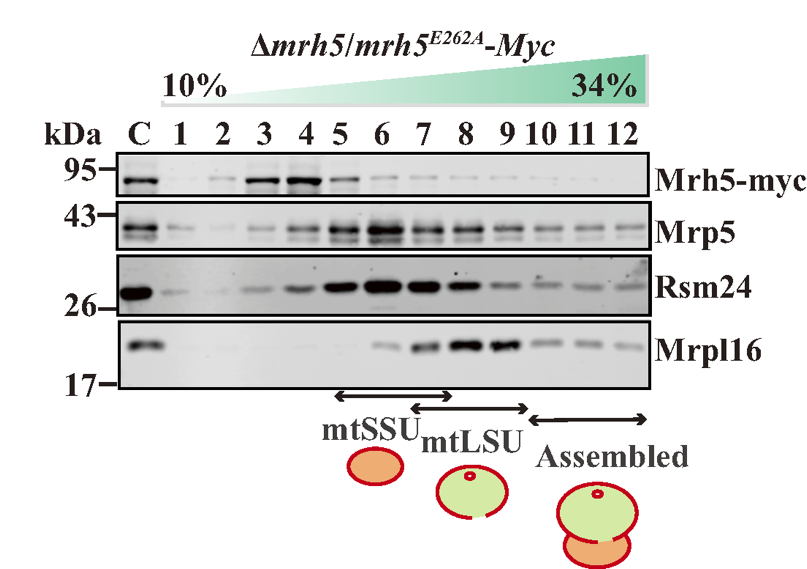


**Fig. S6. The E262A mutation in Mrh5 abolishes its association with mtSSU.** Mitochondrial extracts were prepared from Δ*mrh5* cells bearing integrated *mrh5^E262A^*-*Myc* and subjected to sucrose density centrifugation. The gradient fractions were analyzed by SDS/PAGE and immunoblotting. The positions of the mtSSU, mtLSU and mitoribosome are determined by using the mtSSU marker Mrp5 and mtLSU marker Mrpl16.

Figure S7


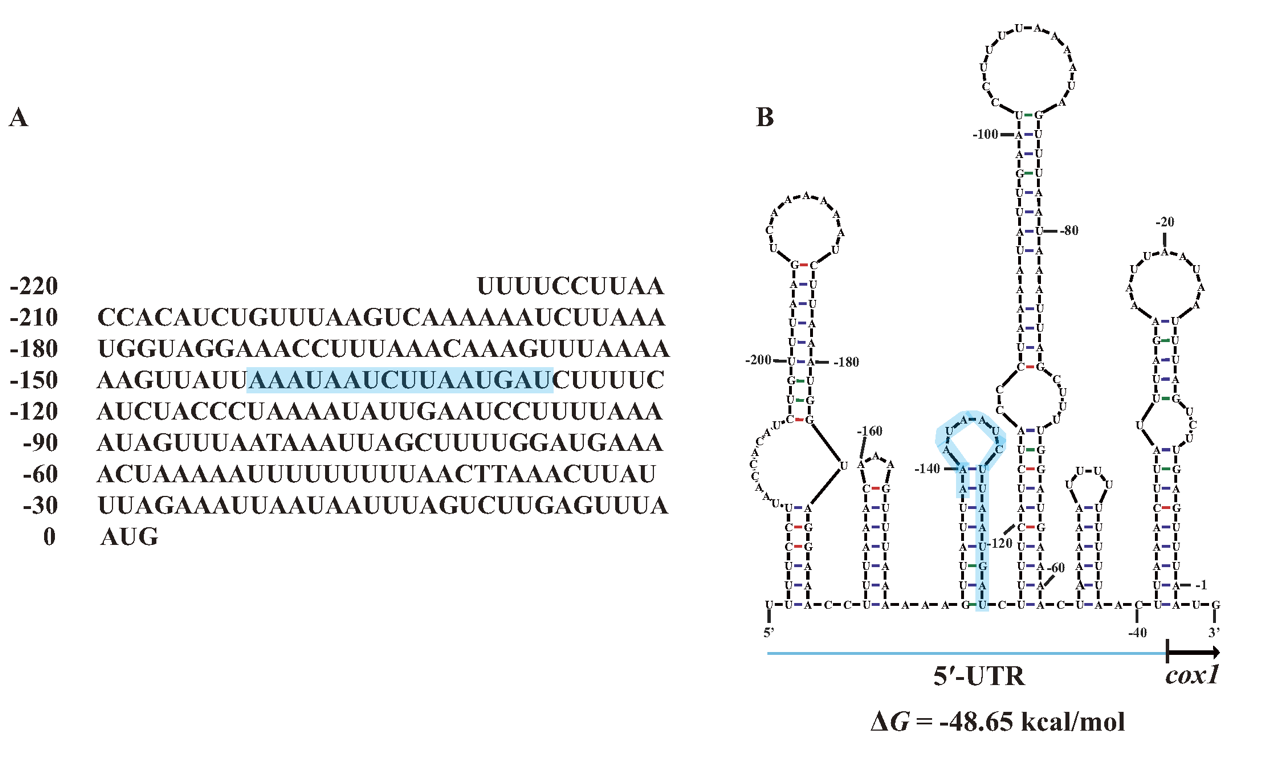


**Fig. S7. Predicted secondary structures of Ppr4 binding sites in the *cox1* 5′-UTR.** (A) The *cox1* 5′-UTR sequence. The 5′-UTR sequence is derived from published literature (56). (B) The secondary structure of the *cox1* 5′-UTR. The RNA structure was predicted using the Mfold web server (57). The predicted Ppr4 binding site in the *cox1* 5′-UTR is highlighted in blue.
